# Conserved syntenic clusters of protein coding genes are missing in birds

**DOI:** 10.1186/s13059-014-0565-1

**Published:** 2014-12-18

**Authors:** Peter V Lovell, Morgan Wirthlin, Larry Wilhelm, Patrick Minx, Nathan H Lazar, Lucia Carbone, Wesley C Warren, Claudio V Mello

**Affiliations:** Department of Behavioral Neuroscience, Oregon Health and Science University, Portland, OR USA; The Genome Institute, Washington University School of Medicine, St. Louis, MO USA; Oregon National Primate Research Center, West Campus, Oregon Health and Science University, Portland, OR USA; Bioinformatics and Computational Biology Division, Oregon Health & Science University, Portland, OR USA

## Abstract

**Background:**

Birds are one of the most highly successful and diverse groups of vertebrates, having evolved a number of distinct characteristics, including feathers and wings, a sturdy lightweight skeleton and unique respiratory and urinary/excretion systems. However, the genetic basis of these traits is poorly understood.

**Results:**

Using comparative genomics based on extensive searches of 60 avian genomes, we have found that birds lack approximately 274 protein coding genes that are present in the genomes of most vertebrate lineages and are for the most part organized in conserved syntenic clusters in non-avian sauropsids and in humans. These genes are located in regions associated with chromosomal rearrangements, and are largely present in crocodiles, suggesting that their loss occurred subsequent to the split of dinosaurs/birds from crocodilians. Many of these genes are associated with lethality in rodents, human genetic disorders, or biological functions targeting various tissues. Functional enrichment analysis combined with orthogroup analysis and paralog searches revealed enrichments that were shared by non-avian species, present only in birds, or shared between all species.

**Conclusions:**

Together these results provide a clearer definition of the genetic background of extant birds, extend the findings of previous studies on missing avian genes, and provide clues about molecular events that shaped avian evolution. They also have implications for fields that largely benefit from avian studies, including development, immune system, oncogenesis, and brain function and cognition. With regards to the missing genes, birds can be considered ‘natural knockouts’ that may become invaluable model organisms for several human diseases.

**Electronic supplementary material:**

The online version of this article (doi:10.1186/s13059-014-0565-1) contains supplementary material, which is available to authorized users.

## Background

**Figure 1:**
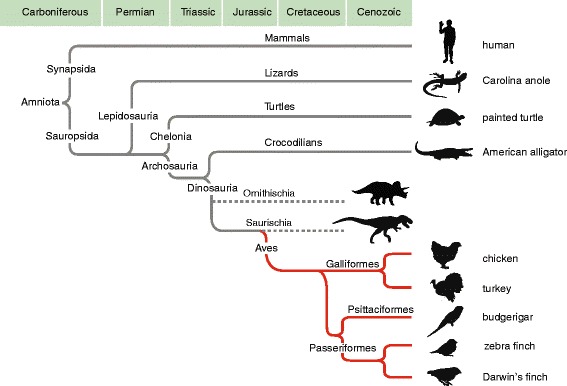
**Schematic depicting the phylogenetic relationships among major Amniote lineages.** Geological eras indicated at the top. Genes that are shared by all organisms shown, but missing in Birds (in red) are presumed to have been lost in non-crocodilian archosaur (possibly Saurischian) ancestors of birds. Names to the right of the silhouettes indicate the extant species analyzed in this study; dashed lines indicate extinct lineages.

Birds are highly successful and diverse descendants of therapod dinosaurs (Figure 1) that have evolved a number of distinct characteristics such as feathers, wings and the ability to fly, a sturdy lightweight skeleton, a toothless beak, high metabolic rate and endothermy, and unique respiratory and urinary/excretion systems that distinguish them from other sauropsids (for example, lizards, turtles, crocodiles [1-3]). To date, however, the genetic basis underlying these traits has been largely unknown. With the recent sequencing and annotation of a large number of avian (60) and sauropsid (5) genomes, including zebra finch, [4], chicken [5], turkey [6], 45 genomes completed in the context of the avian phylogenomics consortium [7,8], 12 additional avian genomes available at NCBI (listed in Methods), Western painted [9] and Chinese soft-shelled turtles, the green anole [10], and the American alligator and saltwater crocodile [11,12], it has become possible to identify genomic features that are unique to birds, and thus possibly associated with the evolutionary emergence of characteristic avian traits.

Avian genomes have been found to be more compact compared to other amniotes. This difference, which correlates with an overall smaller cell size, was speculated to reflect an adaptation related to the higher rates of oxidative metabolism necessitated by the evolution of flight [13,14]. However, more recent evidence for similar genomic streamlining in non-avian dinosaurs, suggests that the evolution of compact genomes may have occurred largely before the emergence of flighted birds [15]. Mechanistically, these reductions in genome size likely occurred as the result of a loss of non-coding DNA sequences, a possibility supported by evidence that avian genomes have less repetitive DNA, fewer pseudogene, and shorter introns compared to mammals [5,16]. Importantly, however, the evolution of avian genomes also appears to have involved a loss of protein coding genes, as the total number of unique identified avian coding genes (for example, 15,508 in chicken according to Ensembl release e71; [17,18]) is considerably smaller than in other tetrapods (20,806 in humans, 18,596 in anole lizard, 18,429 in frogs). Indeed, paralog analysis demonstrates an overall higher occurrence of gene families with fewer members in birds than in other vertebrates [13]. Finally, birds are also known to have high rates of chromosomal rearrangements compared to other organisms, which could in principle have resulted in significant losses of syntenic groups of protein coding genes [5,19].

We have previously observed that analysis of side-by-side chromosomal alignments of 1-to-1 orthologs from representative vertebrate species can be used to identify protein coding genes that are missing in birds [4]. Specifically, we found that a syntenic gene block on mammalian chromosome X that includes Synapsin 1 (*SYN1*) is missing in the genomes of both zebra finch and chicken, but present in lizards. To gain a more comprehensive understanding of the extent of possible avian gene losses, we decided to systematically apply this approach to the entire genome of birds. Specifically, we compared the syntenic arrangements of orthologous genes in the genomes of non-avian sauropsids as well as humans with those of birds, coupled with extensive BLAT/BLAST searches of avian genomes and manual verification of orthology for any resulting hits. Our reasoning was that genes present in non-avian sauropsids and humans but missing in a large number of distantly related birds, including those that were used to define the avian phylogenomic tree [8], likely represent gene losses that are characteristic of the avian lineage, rather than genomic features specific to lizard or to only a few bird species. We found that approximately 274 genes that are present in conserved syntenic blocks or in close proximity to these blocks at discrete chromosomal locations in non-avian sauropsids and mammals are absent in all birds examined. We also found that these genes are for the most part present in crocodilian genomes, indicating that the losses likely occurred within the dinosaur/avian lineage rather than in a more distant archosaur ancestor. A comprehensive bioinformatics analysis revealed that a substantial number of missing genes are associated with lethality or disease phenotypes that affect major tissues, organs, or systems in mice and/or humans. In several cases paralogous genes and/or biochemical or physiological adaptations that are present in birds may have provided compensation for these gene losses. We discuss the possible functional and evolutionary implications of this loss of protein coding genes.

## Results

### Evidence for a large-scale loss of syntenic protein coding genes in birds

Starting with the complete set of gene model predictions from Ensembl (e71), we first conducted a comprehensive comparative genomics analysis to identify orthologous gene sets in humans (*Homo sapiens*), a lizard (green anole; *Anolis carolinensis*) representing a non-avian sauropsid, a galliform, (chicken; *Gallus gallus*) representing a basal avian order with a high quality genome assembly, and an oscine passeriform (zebra finch; *Taeniopygia guttata*; Figure 1). We initially focused on chicken and zebra finch, since these represented the best assembled and curated avian genomes available in Ensembl at the time we began this study. Out of 18,596 protein coding genes in lizard, 12,113 are predicted to have 1-to-1 orthologs in humans. Of these, only 10,554 also have 1-to-1 orthologs in chicken and/or zebra finch, thus revealing a total of 1,559 genes that are potential candidates for missing genes in birds (Table 1A). We next aligned side by side the entire set of 1-to-1 orthologs between humans and lizards based on chromosomal location with the corresponding orthologs from birds to search for cases where conserved genes in humans and lizards were missing in both avian species. In several cases we also examined the corresponding regions in the Painted turtle genome (*Chrysemys picta bellii*), to help establish synteny in regions that are poorly assembled in the lizard genome. We found that 537 out of the 1,559 putative missing genes in birds cluster into approximately 100 conserved syntenic blocks in lizard and humans. The approximately 1,000 remaining candidate missing genes occur as singletons throughout non-avian genomes, or are associated with segments that have not been included in the main avian assemblies (that is, Chr_Unk; see Methods for details). It is thus not possible to conclusively establish orthology, or whether these other missing genes are true singletons or part of syntenic blocks. In contrast, the missing syntenic gene blocks are relatively large (typically >80,000 bps), thus their absence can be verified with high confidence in a high quality genome like that of the chicken. We decided to focus our efforts on these missing blocks, as it is less likely that they are present in unsequenced or unassembled segments of avian genomes.

**Table 1:** **Evidence for a loss of protein coding genes in birds**

**A. Identification of protein coding gene orthologs in lizard, human, chicken, and zebra finch** ^**a**^	
**Genes/Models**	**Category**
21,122	Lizard gene models (Ensembl; AnoCar2.0)
18,596	Lizard protein coding gene models
12,113	Lizard models with 1-to-1 or apparent 1-to-1 orthologs in humans (GRH37)
10,554	Lizard models with 1-to-1 or apparent 1-to-1 orthologs in chicken (WASHUC2) and/or zebra finch (taeGut3.2.4)
**1,559**	Lizard models with no apparent orthologs in birds
**B. Confirming gene loss in missing syntenic blocks in birds**	
537	Initial set of candidate avian missing genes that are present in human/lizard syntenic blocks
+25	Additional candidate missing genes that were not predicted by Ensembl, but were identified in lizard genome (‘no model’ entries on Tables S1A and S6)
+50	Additional candidate missing genes with incorrectly annotated lizard model (‘†’ entries on Table S1)
- 89	Genes found in birds based on Entrez gene, RefSeq, and cloned mRNA databases (Table S4A)
- 75	Genes found in birds based on lizard/human mRNA and protein BLAT/BLAST searches of avian genomes, trace archives, and EST/mRNA databases (Table S4B)
−174	Genes found in birds based on alligator/lizard/human protein tBLASTn searches of 60 avian whole genome shotgun contigs or evidence based on Refseqs in these species (Table S6)
**274**	Final set of avian missing genes (Table S1)
**C. Final breakdown of curated set of genes missing in birds**	
162	Genes that are part of missing syntenic blocks (Table S1A)
112	Genes that are in close proximity to missing syntenic blocks (Table S1B)
**274**	Total avian missing genes

^a^Ensembl release e71.

**Figure 2:**
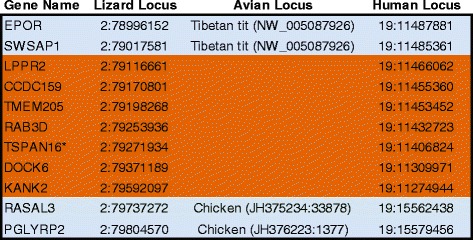
**Evidence for avian genes missing in syntenic blocks.** Example of avian missing syntenic block, revealed by local chromosomal alignment of 1-to-1 orthologous genes in lizard, chicken, and humans, based on chromosomal position in lizard (for full set of deletions see Additional file 1: Table S1A). Syntenically ordered genes missing in birds are shaded in orange; flanking genes that are shared by all species including birds (for example, chicken, Tibetan tit) are shaded in blue. The relative position of each individual gene locus is indicated by chromosome number (for example, chr2, 19) and starting base of the corresponding Ensembl gene model. The asterisk indicates a gene with no predicted Ensembl model in lizard; its location was determined by cross-species BLAT-alignment with sequence from the lizard ortholog.

We next conducted exhaustive verification steps (see Methods) to confirm that the genes in the identified syntenic blocks are indeed missing in birds. This effort (summarized in Table 1B) corrected a large number of misannotated gene predictions and cloned mRNAs in birds, while identifying several previously unknown orthologs and paralogs. First, to our initial list we added 25 genes that were not predicted by Ensembl in lizard. Based on curation of other databases (for example, RefSeqs) and/or cross-species BLAT alignments, we found that these genes are truncated by sequence gaps but are present in the correct synteny in lizard (for example, *TSPN16* in Figure 2; ‘no model’ entries in lizard in Additional file 1: Table S1). We also added 50 genes that have an unannotated lizard Ensembl model but whose correct orthology could be established based on synteny (Additional file 1: Table S1 gene models indicated by a ‘†’). Next, we found that 28 genes on our missing list have avian entries in Entrez Gene, Ensembl, or RefSeq but these are misannotated, corresponding instead to a related family member (Additional file 1: Table S2); 14 of these represent close but previously uncharacterized paralogs (Additional file 1: Table S3). Next, we removed 89 genes from our missing list that were not predicted in avian genomes by Ensembl, but that we found to be present in birds based on a manual verification of entries in Entrez Gene, RefSeq or NCBI avian mRNAs (Additional file 1: Table S4A). We also removed 75 genes that were not previously described in birds, but that we found to be present based on BLAT or BLAST searches of avian genome assemblies (including turkey, medium ground finch, and budgerigar), or avian EST databases (Additional file 1: Table S4B). In most cases these results provide a first demonstration of the existence of these gene in birds. In contrast, 114 genes in our missing list gave significant hits in cross-species BLAT searches of the chicken or zebra finch genomes using the lizard Ensembl gene models as queries, however all hits were to related gene family members (Additional file 1: Table S5) or to close paralogs (Additional file 1: Table S3).

Lastly, we found that a subset (174) of our candidate avian missing genes are present in one or several avian genomes recently assembled and submitted to NCBI, including those sequenced as part of the Avian Phylogenomics Consortium; the evidence derives from extensive further curation of RefSeqs (Additional file 1: Table S6A) and tBLASTn searches of WGS databases (Additional file 1: Table S6B). Of note, the analysis included a ratite (ostrich), indicating that this gene subset is also largely present in basal paleognaths. For two genes, *CATSPERB* and *CCAR12*, the only evidence for their presence among birds comes from a ratite, suggesting that they were likely present in basal paleognaths and possibly lost in modern neognaths. We also note that for several genes in this subset, direct confirmation of orthology was not possible as the hits were to segments that do not allow synteny verification. We took, however, a conservative approach and removed from our avian missing gene list all genes that had a significant avian hit that preferentially cross-aligned back to the correct ortholog in a non-avian species (that is, reciprocal best alignment criterion). It is important to note that this subset of genes (Additional file 1: Table S6) cannot be found in the chicken genome. The chicken assembly we have analyzed (galGal4; 2011) is currently the best-assembled and most completely sequenced avian genome, with much shorter and fewer gaps and thus more complete than the version described in Hillier *et al*. [5]. Accordingly, this latest assembly contains the orthologs for many conserved genes that could not be found in the previous assembly (for example, [20]), and yielded significant BLAT-alignments for approximately 96% of genes from a positive control search set consisting of randomly selected lizard gene models with known orthologs in birds). Lastly, this subset found in other avian species cannot be found in the chicken transcriptome databases. These observations suggest that chicken (or possibly galliformes) may have undergone further syntenic gene losses compared to other birds. As our main goal was to identify genomic losses shared by all birds, these genes are not considered further here and will be the focus of future studies.

Out of these efforts, we determined with high confidence that 274 genes are missing in birds. Of these, 162 are clustered in blocks that have identical arrangements in lizard and humans (example in Figure 2; full list in Additional file 1: Table S1A). Altogether, these avian missing blocks amount to 7.42 and 3.92 Mb in humans and lizard, respectively (see Methods for details). The other 113 confirmed avian missing genes are in close proximity to these syntenic blocks (full list in Additional file 1: Table S1B). All these genes currently have no corresponding entries in any avian database.

It is important to note that we used permissive search filters followed by extensive manual verification. Furthermore, the successful search of the chicken assembly with a control gene set comprised of randomly selected genes that are 1-to-1 orthologs in lizard and human, are present in birds, indicating that the use of lizard models and the settings and criteria in our cross-species searches was adequate and sensitive to detect the corresponding orthologs, if present, in a well assembled avian genome. We also note that the lizard and human models used in cross-species alignments readily identified crocodilian orthologs (see also section on crocodilians below), even in cases where models had low conservation (for example, orthologs that failed to cross align or that have low percent sequence identity when comparing lizard and humans). Nonetheless, to minimize the concern that we might have missed genes due to low sequence conservation, our searches for low conservation genes in avian WGS databases used queries from multiple species, including from crocodilians. Lastly, we note that 19 genes in our curated missing set have been previously reported as missing in different bird species by independent searches of the genome databases or by a variety of molecular or protein biochemistry methods (Additional file 1: Table S7), lending further support to the validity of our curated list of avian missing genes.

To further rule out the possibility that we might not be detecting some genes because they are short and fast diverging (that is, low conservation when comparing orthologs across vertebrate groups) we conducted further analyses to compare the relative distributions of the lengths of coding sequences (CDS) for genes in the avian missing gene set versus those derived from the entire set of lizard genes present in birds). We found that the size distributions are similar in shape and do not differ significantly (two-tailed ANOVA with log-normalized values; *P* =0.3; Additional file 3: Figure S2A). Moreover, the relative percentages of short genes (that is, genes <500 bp) are nearly identical across the two gene sets (9% vs. 10% for the missing vs. present gene sets). In addition, we found no significant relationship between the sizes of the avian missing genes and the amino acid % identity of the predicted proteins when comparing the human vs. lizard orthologs (Additional file 3: Figure S2B). Thus, there appears to be no obvious bias in the missing gene set towards either smaller (or larger) genes, or towards small genes that are highly divergent in regards to protein sequence.

### Syntenic gene losses localize to discrete chromosomal sites

**Figure 3:**
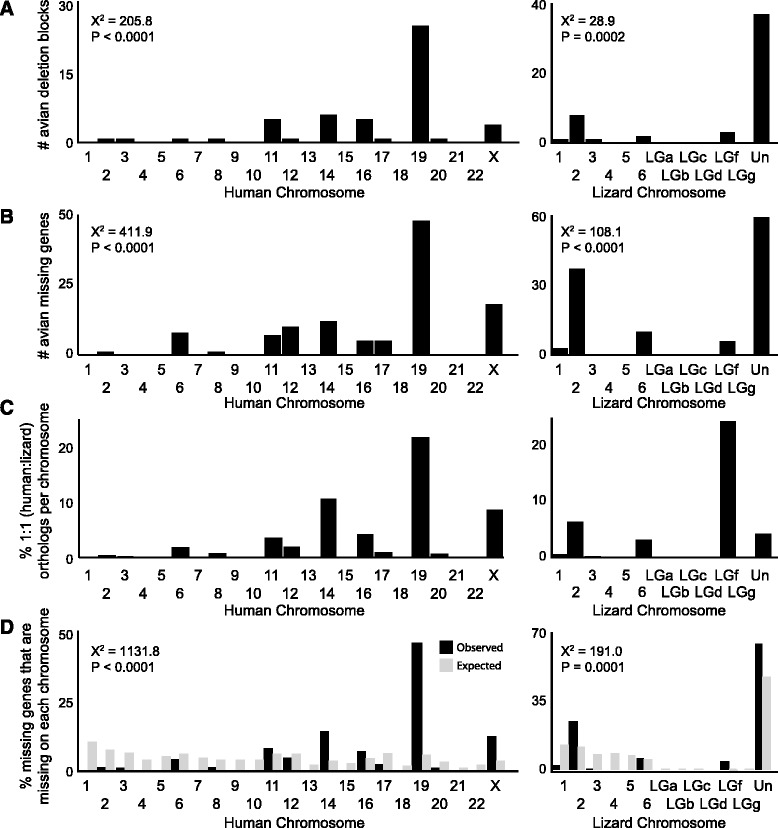
**Distribution of avian missing genes and syntenic blocks on human and lizard chromosomes. (A)** Plots showing the numbers of avian missing syntenic blocks present on human (left) and lizard (right) chromosomes. In both species, the observed number of deletions per chromosomes is significantly different from a predicted uniform distribution based on chromosome size (Chi-squared test for independence). **(B)** Plot showing the numbers of missing genes on human (left) and lizard (right) chromosomes. In both species, the observed number of deletions per chromosomes is significantly different from a predicted uniform distribution based on chromosome size (Chi-squared test for independence). **(C)** Plot showing the percentage of missing genes, normalized by the number of 1-to-1 (lizard-to-human) orthologs, on each chromosome. **(D)** Plot showing the percentage of missing genes, normalized by the total number of missing genes on each chromosome in human (left; black bars) and lizard (right; black bars). Gray bars indicate the relative percentage of deletions for each chromosome that would be expected if the deletions occurred at according to a random distribution similar to the loss of avian missing genes in blocks and as singletons (see Methods for details). Lizard microchromosome LGe was excluded from the analysis because it contained coding genes predictions. Abbreviations: Un, unplaced segments in lizard genome.

The genes we found to be missing in birds are not uniformly distributed across the genomes of non-avian species, but instead are concentrated in a small number of chromosomes. This asymmetry is clearly seen when plotting the number of missing syntenic blocks per chromosome (Figure 3A), and does not simply reflect differences in chromosome size. Instead, the distribution is significantly different from what would be expected if the blocks were uniformly distributed among the chromosomes according to chromosome size (X^2^ = 205.8; df = 22; *P* <0.0001 for human and X^2^ = 28.9; df = 12; *P* <0.0002 for lizard). In fact, only five of the 23 human chromosomes (chr19, X, 11, 14 16), and two of the 12 lizard chromosomes (chr2, and LGf) have a greater number of deletion blocks than would be expected by chance, whereas the majority of the other chromosomes have fewer blocks than expected. In particular, human chr19, a very gene-rich chromosome, contains the majority of the missing blocks, despite being one of the shortest human chromosomes. A similar asymmetric distributions was observed by plotting the number of avian missing genes per chromosome (Figure 3B); this distribution differs from what would be expected based on the total numbers of genes present on each chromosome (X^2^ = 411.9; df = 22; *P* <0.0001 for human and X^2^ = 108.1; df = 12; *P* <0.0001 for lizard). We also see an asymmetry when plotting the number of avian missing genes per chromosome relative to the total number of 1-to-1 lizard/human orthologs on each chromosome (Figure 3C), or by plotting the number of missing genes per chromosome normalized by the total number of missing genes (Figure 3D; black bars). This latter distribution differs significantly from what would be expected from a randomly selected subset of genes derived from the 1:1:1 chicken:human:lizard ortholog set (Figure 3D; gray bars, n = 274 genes; X^2^ = 1131.8; df = 22; *P* <0.0001 for human and X^2^ = 191.0; df = 12; *P* <0.05 for lizard). Again this analysis demonstrates the large contributions of human chr19 and lizard chr2. One can also note in lizard the large relative contribution of chrLGf, a microchromosome that contains only a small number of genes, and that a large number of avian missing blocks and genes localize to contigs that are unplaced in the current assembly (Figure 3A-D, right panels).

**Figure 4:**
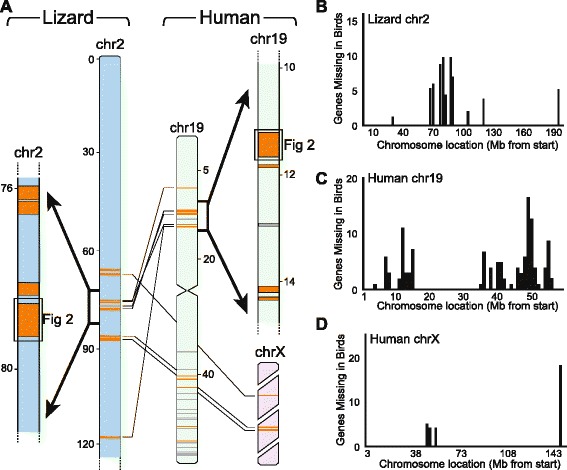
**Evidence for avian gene deletion domains on lizard and human chromosomes. (A)** Chromosome maps illustrating the distribution of avian missing blocks on lizard chr2 and human chr19 and X. The thin lines indicate locations of corresponding avian missing gene blocks (in orange); arrows indicate expanded segments for detailed views (orange shading denotes block inclusion criteria as in Additional file 1: Table S1, gray blocks denote human blocks that are on unplaced contigs in lizard). The boxed segments in the expanded views refer to a missing block example presented in Figure 2. **(B**-**D)** Plots of the numbers and positions of avian missing genes along chromosome 2 in lizard, and human chromosomes 19 and X reveal their localization to ‘hot spots’.

Interestingly, the avian missing genes are also non-uniformly distributed along the length of each respective chromosome. In fact, most of the deletions cluster within small segments or domains, as visualized by mapping the locations of missing blocks on their respective chromosomes (Figure 4A), or by plotting the number of missing genes according to chromosomal location (examples on Figure 4B-D). When comparing the relative positions of the missing blocks in lizard vs. humans one notices that these represent chromosomal regions that appear to have undergone extensive rearrangements between these organisms (Figure 4A). To investigate this issue further, we aligned the complete set of 1-to-1 orthologs according to human chromosome location, identified in humans the gene blocks immediately flanking each avian missing block, and examined in birds the relative positions of these flanking blocks (see Methods for details). Considering 17 individual missing blocks for which we could assign flanking blocks in birds to known chromosomal locations (i.e. not chr_Unk), we found that the majority (n = 10) were present on different chromosomes in birds (example on Additional file 2: Figure S1A), and the remaining seven were located on the same avian chromosome, but the flanking blocks were out of order, in reverse order, or several megabases apart in comparison to their location in humans (Additional file 2: Figures S1B and S1C) and/or lizards (not shown). These results indicate that most of the avian missing syntenic blocks are located in chromosomal regions that appear to have undergone significant inter- and intra-chromosomal rearrangements when comparing humans and birds.

**Figure 5:**
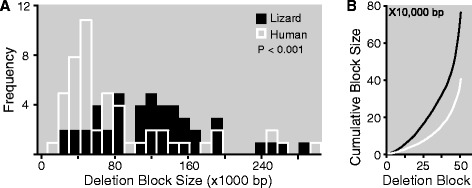
**Analysis of avian deletion block sizes on lizard and human chromosomes. (A)** Frequency distributions of missing blocks arranged by size reveal deletion block sizes are significantly larger on average in lizard (black bars) than human (white bars; Pair-wise Wilcoxon Signed Ranks test). The sizes of each blocks corresponds to the minimal size of the human and lizard chromosomal segments containing all the orthologs within each of the avian missing syntenic blocks. **(B)** Cumulative sum of avian deletion block sizes in lizard (black) and human (white). Only blocks of three or more genes presented on Additional file 1: Table S1A are included in the analysis. Abbreviations: bp, base pairs.

We also found that the average size of the avian missing syntenic blocks is almost twice as large in lizards as compared to humans (142.7 ± 20.2 vs. 75.6 ± 12.4 Kb; Mean ± SEM; Wilcoxon matched-pairs signed-ranks test; *P* <0.001), a difference that can be observed when plotting side by side the size distributions of the syntenic blocks in the two species (Figure 5A). Reflecting this difference, the cumulative size of the missing segments in the lizard genome is also considerably larger than that observed in humans (7.42 vs. 3.92 MB; Figure 5B). Furthermore, this species difference is largely due to size differences in avian missing syntenic blocks that occur on just a few human chromosomes, including 19 (lizard vs. human average block sizes for human chr19: 150.0 ± 72.3 vs. 84.9 ± 21.6 Kb, *P* <0.001; Wilcoxon matched-pairs signed-ranks tests). Consistent with this finding, of the approximately 4 MB of human genomic DNA that corresponds to the missing syntenic blocks in birds, approximately 90% is derived from combined losses on chr19 (54.0%), X (9.2%), 11 (9.1%), 14 (9.1%), and 16 (7.5%).

### Estimating gene loss in avian ancestors

We next searched for the 274 genes confirmed to be missing in birds in two recently available crocodilian genomes, the American alligator (*Alligator mississippiensis*) and saltwater crocodile (*Crocodylus porosus*) [8,11]). To establish a baseline, we first reasoned that genes present in birds, lizard, and humans are also highly likely to be present in crocodilians. To test this prediction, we performed BLAT-alignments with our control set of lizard gene models with known orthologs in chicken (that is, positive control gene set) to the alligator genome. We found that 91% of these genes yielded significant hits to alligator or crocodile. Next, we BLAT-aligned the entire set of 274 lizard gene models corresponding to avian missing genes to both the alligator and crocodile genomes. We found significant hits in alligator and/or crocodile (Additional file 1: Table S8) for 154 (approximately 56%) queries. More recently, as RefSeq annotations have become available for crocodilians, we searched the remaining genes on our avian missing gene list, and manually verified the presence of another 83 genes, at the correct synteny. Thus the vast majority (approximately 86%) of the avian missing genes are present in the current crocodilian assemblies. While we have found no convincing evidence for the remaining 38 genes, we note that these assemblies are still largely incomplete with significant gaps and low quality regions, thus it is very likely that we are underestimating the presence of avian missing genes in crocodiles.

To better understand the uniqueness of these gene losses among vertebrates, we further examined the orthology of the avian missing gene set based on a detailed analysis of Ensembl gene models. While by definition 100% of these genes are present in non-avian amniotes (that is, humans and lizard), we found that fully approximately 94% to 95% are present in sarcopterygians (that is, coelacanth, *Xenopus*), and approximately 90% are present in teleosts (zebra fish, fugu). Thus, the majority of missing avian genes are conserved, and were likely present in a fish ancestor. Moreover, we found that only a small subset of the avian missing genes were also lost in any of the non-amniote vertebrate lineages, including approximately 1% in fish, approximately 3% in coelacanth, and approximately 10% in *Xenopus* - the latter likely being an overestimate due to the relatively poor quality of genome assembly and predictions. Most importantly, rather than occurring as clusters in syntenic blocks, all of these losses appear to be distributed throughout the respective genomes. Thus, the extensive loss of genes in syntenic blocks appears to have been a unique phenomenon that occurred only in birds, or within an archosaur organism within the dinosaurian/avian lineage ancestral to extant birds.

### Bioinformatics analysis of avian missing genes

**Figure 6:**
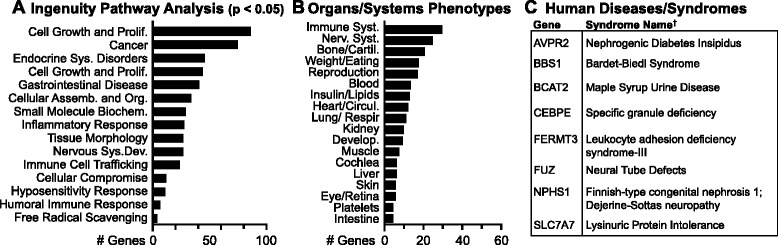
**Bioinformatics analysis of avian missing genes.** Breakdown of avian missing genes according to their association with: **(A)** The Ingenuity Pathway Functional enrichment categories related to Diseases and Disorders, Molecular and Cellular Functions, and Physiological System Development and Function (*P* <0.05; ranked according to number of genes), **(B)** organ and/or systems phenotypes associated with mouse knockout studies, and/or **(C)** genetic diseases and syndromes in humans that are also lethal when knocked out in mice. Further details are presented in Additional file 1: Tables S9-S13. * - includes both apoptotic and non-apoptotic; ** - includes clathrin related and receptor clustering; *** - includes kinases, phosphatases, and calmodulins, **** - includes glycosylation, methylation, and acetylation; † − [21].

We next conducted bioinformatics analyses to assess the potential functional impact of the observed gene losses on the dinosaur/avian lineage. A functional enrichment analysis of the missing gene set using Ingenuity Pathway Analysis (IPA; see Methods for details) revealed a range of biological function categories that are significantly enriched (*P* <0.05; Figure 6A and Table 2A). This included clusters of genes associated with functional categories such as inflammatory response and gastrointestinal disease, molecular and cellular functions such as free radical scavenging, and physiological system development and function such as tissue morphology, humoral immune response, and immune cell trafficking (Table 2A). Further analysis of enriched specific functional categories revealed that many of the missing genes participate in major cellular functions and/or are implicated in a severe human hereditary diseases and disorders (Additional file 1: Table S9). This included genes associated with cell growth and proliferation, hereditary disorders such as X-linked mental retardation, leukocyte adhesion deficiency types I and III, X-linked spinocerebellar ataxia type 1, hematological system development and function, immune function, and nervous system development. The missing avian genes are also enriched in a number of canonical pathways that regulate the functions of a wide array of organs and signaling systems. The most significant pathways (n = 22; *P* <0.005) are presented in Table 2B, and include protein kinase A signaling, G-protein coupled receptor signaling, T cell receptor and anergic T lymphocyte regulation, estrogen-dependent breast cancer and GNRH signaling, as well as pathways related to cardiac hypertrophy and melanocyte development to name a few.

**Table 2:** **Enriched functional categories and canonical pathways for genes missing in birds**

**A. Enriched functional categories for genes that are missing in birds**			
**Rank** ^**a**^	**Enriched functional category**	***P*** **value range**	**No. of genes**
**Diseases and Disorders**			
1	Cancer	3.03E-03 - 3.69E-02	74
2	Endocrine system disorders	3.03E-03 - 3.69E-02	45
3	Gastrointestinal disease	4.59E-04 - 3.56E-02	40
4	Inflammatory response	1.54E-04 - 3.69E-02	27
5	Hypersensitivity response	3.08E-04 - 3.69E-02	10
**Molecular and Cellular Functions**			
1	Cellular growth and proliferation	2.11E-04 - 3.69E-02	85
2	Cellular assembly and organization	7.68E-04 - 3.69E-02	33
3	Small molecule biochemistry	2.14E-04 - 3.69E-02	28
4	Cellular compromise	3.08E-04 - 3.69E-02	11
5	Free radical scavenging	2.14E-04 - 3.69E-02	3
**Physiological System Development and Function**			
1	Hematological system development and function	1.54E-04 - 3.69E-02	43
2	Tissue morphology	1.54E-04 - 3.69E-02	26
3	Nervous system development and function	7.68E-04 - 3.69E-02	26
4	Immune cell trafficking	8.09E-04 - 3.69E-02	23
5	Humoral immune response	8.09E-04 - 1.73E-02	6
**B. Enriched canonical pathways for genes that are missing in birds (** ***P*** **<0.005)**			
**Rank** ^**b**^	**Ingenuity canonical pathway**	***P*** **value** ^**c**^	**No. of genes**
1	Protein kinase A signaling	1.10E-03	13
2	Cardiac hypertrophy signaling	5.01E-04	10
3	G-Protein coupled receptor signaling	4.90E-03	9
4	Cellular effects of sildenafil (Viagra)	2.14E-04	8
5	Estrogen-dependent breast cancer signaling	1.05E-03	8
6	Sertoli cell-Sertoli cell junction signaling	1.78E-03	8
7	Calcium signaling	1.78E-03	8
8	PI3K signaling in B lymphocytes	1.12E-03	7
9	GNRH signaling	1.17E-03	7
10	T cell receptor signaling	1.35E-03	6
11	fMLP signaling in neutrophils	2.34E-03	6
12	Gαs signaling	2.45E-03	6
13	14-3-3-mediated signaling	3.47E-03	6
14	Melatonin signaling	1.78E-03	5
15	FLT3 signaling in hematopoietic progenitor cells	2.29E-03	5
16	Acute myeloid leukemia signaling	2.75E-03	5
17	Regulation of IL-2 expression in activated and anergic T lymphocytes	3.09E-03	5
18	Melanocyte development and pigmentation signaling	3.98E-03	5
19	α-Adrenergic signaling	4.68E-03	5
20	Bladder cancer signaling	4.68E-03	5
21	UVA-induced MAPK signaling	4.90E-03	5
22	Polyamine regulation in colon cancer	2.45E-03	3

^a^Ranking by number of avian missing genes within each enrichment category type.

^b^Ranking by number of avian missing genes associated with the pathway.

^c^The complete list of functions associated with these categories is presented in Additional file 1: Table S9.

To examine whether these cellular functions and/or pathways are specific to the avian missing genes or more generally associated with any set of genes of comparable overall size and syntenic organization, we performed a parallel IPA on control gene sets (see Methods for details). These control sets represent a reasonable expectation for the range of phenotypic enrichments that might be expected (that is, null expectation) if a set of losses occurred in randomly deleted syntenic gene clusters located on the same chromosomes as the missing gene set. When we compared broad enrichment categories we found that several were shared with those seen for the missing gene set (listed in Table 2A), including cancer, inflammatory response, and endocrine system disorders. However, careful examination of specific functional categories revealed remarkably few overlaps. In fact, of the top 58 categories found in the missing genes set (as established by a *P* <0.01 cutoff; Additional file 1: Table S9), only one - X-linked mental retardation - was also present in one of the control sets at the same cutoff, thus suggesting that the majority of these annotations are specific to the missing gene set. Similarly, when we compared the combined set of significantly enriched (*P* <0.005) canonical pathways associated with either of the control genes set with the top canonical pathways enriched in the missing gene set (listed in Table 2B), we found no overlapping pathways, thus further indicating that many of the pathways that are predicted to be associated with the loss of the avian missing gene set are uniquely associated with the missing gene set.

Studies characterizing the effects of spontaneous, induced, or genetically-engineered mutations provide the best inferential kind of evidence for understanding the potential impact of gene losses. We therefore retrieved from the Mouse Genome Informatics (MGI) [22] database the sets of phenotypes that have been observed in rodents in association with manipulations of the avian missing genes. This included cases where just a single knockout was sufficient to cause the phenotype, as well as others where multiple knockouts were required. We then classified the retrieved entries according to affected tissues, organ, and systems, adding genes based on searches of Entrez Gene and the scientific literature (for example, PubMed, Google Scholar). This analysis revealed that 98 genes are associated with at least one phenotype that affects a major organ and/or system, including the central and peripheral nervous systems, the immune system, bone and cartilage, the reproductive system, lungs and respiration, and regulation of weight and appetite (Figure 6B; Additional file 1: Table S10A); a subset of these phenotypes is only present when genes are knocked out together with other related genes. Interestingly, a small number of avian missing genes (approximately 5%) are related to mouse phenotypes associated with tissues and/or organ functions that are absent in birds, including hair, teeth, placenta, and lactation (Additional file 1: Table S11). Finally, 43 of the missing genes are associated with a lethal phenotype in mice, including partial and complete embryonic or perinatal lethal, or premature death. Of these, 27 have a lethal phenotype when individually knocked out (Additional file 1: Table S12A), and 16 are only lethal when knocked out in combination with one or more additional genes (Additional file 1: Table S12B).

Since a large number of the avian missing genes are associated with a severe and/or lethal phenotype in mice, we wondered whether these associations are unique (that is, non-random) with respect to the genes missing in birds, or more generally associated with any comparably sized and organized sets of genes. To address this question we applied a permutation analysis, and performed MGI phenotype classification on 1,000 independent control gene sets (see Methods for details). We found that the number of genes associated with a mouse phenotype in the missing gene set (n = 98, excluding ‘no abnormal phenotype detected’) is significantly smaller than would be expected based on an analysis of the permutation dataset (Additional file 4: Figure S3A; two-sided permutation test; *P* = 0.021). We also compared the number of genes associated with each phenotype in both gene sets, and found 11 phenotypes that are significantly under- or over-enriched in the missing gene set (two-tailed permutation test with Benjamini-Hochberg false discovery rate (FDR) correction for multiple comparisons; *q* <0.05). The complete list of mouse phenotypes for which the number of expected and observed genes differed by at least one gene is presented in Additional file 1: Table S10B. Among the most significant phenotypes are those associated with body weight and energy metabolism (that is, MP:0003960, increased lean body mass; MP:0009289, decreased epididymal fat pad weight; MP:0010400, increased liver glycogen level), immune function (that is, MP:0008050, decreased memory T cell number; MP:0008765, decreased mast cell degranulation), and lung function (that is, MP:0010809, abnormal Clara cell morphology; MP:0011649, immotile respiratory cilia). We also found a strong trend (two-tailed permutation test without FDR correction; *P* <0.05) towards a greater association of genes with phenotypes related to lethality, including premature death and complete embryonic lethality, in the permutation set as compared to the avian missing gene set (Additional file 1: Table S10B). Lastly, a broad range of phenotypes are numerically, but not statistically different, or occur with similar frequency in both groups. These correspond to phenotypes that would be generally associated with the loss of similarly sized and organized sets of genes in other regions of the chromosomes of amniotes (Additional file 5: Figure S4).

Since the IPA provided suggestive evidence that many gene losses are associated with severe hereditary diseases in humans, we next consulted the Online Mendelian Inheritance in Man (OMIM) [23] and conducted further keyword searches in Entrez Gene. We found that a total of 32 genes are associated with a specific genetic disorder or syndrome in humans. We then verified each OMIM entry and classified cases where the disease was associated with the loss of a gene or gene function (Additional file 1: Table S13A), or caused by a gain of function mutation (Additional file 1: Table S13B). In most cases the loss of function mutations were associated with autosomal recessive disorders, but also included cases of X-linked disorders or autosomal dominant haploinsufficiency. Importantly, a subset of the genes linked to human disorders is also associated with a lethal phenotype in mice (Figure 6C). Given the severity of many of these diseases we wondered whether the observed set might contain fewer OMIM disease terms than would expected by chance. Such a finding would be consistent with the hypothesis that gene losses associated with highly deleterious phenotypes are less likely to be tolerated and thus will be less frequent in genes that are actually missing in birds than in control sets. Indeed, when we compared the number of OMIM disease terms that are associated with the 1,000 permutations of control sets versus the missing gene set, we found that the missing gene set contained significantly fewer OMIM disease terms than would be expected by chance (two-sided permutation test; *P* <0.001; Additional file 4: Figure S3B). We also note that, as might be expected, although the control gene sets were associated with a wide range of severe disease phenotypes, we did not find any cases where a specific disease term that was associated with a missing gene was also associated with a gene from the control sets. Thus, we conclude that the set of disease traits associated with the identified avian missing genes is both specific and non-random.

Since the disease phenotypes (and lethality) associated with the gene disruptions in mammals did not obviously align with known avian traits, we hypothesized that perhaps the genetic background of birds was capable of providing compensation for the avian missing genes. Evidence for compensation, if found, would be of interest since it would indicate that compensatory genetic or functional mechanisms might underlie avian adaptations, and also suggest possible treatments or cures for lethal and morbid conditions in humans. To explore this possibility we conducted a comparative functional enrichment analysis (Blast2GO) [24] in order to compare the impact of the loss of the same set of avian missing genes against the genetics backgrounds of chicken, humans, and lizard. We first identified the set of enriched GO terms associated with the avian missing genes compared to the entire universe of extant protein coding genes for each of the species analyzed. We reasoned that GO term enrichments in a given organism reflect functions that are over-represented in the missing gene set compared to the genetic background of that organism, and thus are likely not functionally compensated within that genetic background; we note that for lizard and humans the analyses were for hypothetical deletions. We found statistically significant (*P* <0.05) GO enrichments in all three genomic contexts, but also found that fewer overall GO enrichments (that is, all GO terms associated biological processes or molecular functions) were associated with the analysis in chicken (n = 235) than comparable analyses in humans (n = 294) or lizard (n = 338). These differences do not reflect obvious biases in either the proportion of BLASTp annotated sequences (85.5, 85.9%, and 77.3% for chicken, lizard, and human, respectively), or in the average number of GO terms that could be assigned to each gene by Blast2GO (7.9 vs. 7.6 vs. 10.2 for chicken, lizard, and human).

**Figure 7:**
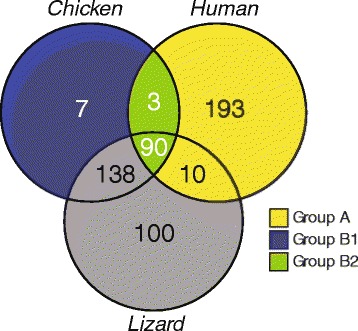
**Assessing the functional impact of the avian missing genes in the context of the chicken, human, and lizard genomes.** Comparative functional enrichment analysis was used to compare the impact of the same set of avian missing genes against the genetics backgrounds of chicken, humans, and lizard. Gene Ontology (GO) term enrichments for pairwise comparison were identified by Fisher’s test (*P* <0.05), and a Venn diagram was used to compare the GO term enrichments and identify: Group A, terms significantly enriched in non-avian species but not in birds (yellow panels), representing functions/pathways that might be disrupted only if the gene loss were to have occurred in non-avian organisms; Group B_1_, terms enriched in birds only (dark blue), representing functions/pathways that might be affected only in the context of avian genomes; Group B_2_, enriched terms shared between birds and humans and/or lizard (green), representing pathways that would be affected in multiple or all species, and for which there are no apparent compensations in these species; and Group C, terms enriched in chicken and lizard (gray), representing functions that would in principle be affected in sauropsids but likely not in mammals, possibly due to compensation in the latter.

We next compared the resulting GO term enrichments across species, to separate organism-specific enriched terms representing functions that are likely to be disrupted only in one given lineage from shared enriched terms representing functions likely to be disrupted in multiple lineages. This analysis identified three groups of terms (detailed in Additional file 1: Table S14): Group A, were significantly enriched in non-avian species (Figure 7, yellow in the Venn diagram), representing functions/pathways that might be disrupted only if the gene loss were to have occurred in non-avian organisms. This group is of particular interest since it identifies functional terms where the corresponding gene loss may have been compensated by the genetic background of birds. Group B, were significantly enriched in birds, where the gene loss would likely not have been compensated by the genetic background of birds. We subdivided these further into terms enriched in birds only (Group B_1_: Figure 7, dark blue), likely representing functions/pathways that might be affected only in the context of avian genomes, and enriched terms shared between birds and humans and/or lizard (Group B_2:_ Figure 7, green), likely representing pathways that would be affected in multiple or all species, and for which there are no apparent compensations in these species. Group C, were enriched in chicken and lizard or lizard only but not humans (Figure 7, gray) representing functions that would in principle be affected in sauropsids but likely not in mammals, possibly due to compensation in the latter. A. We analyzed Groups A and B further (Additional file 1: Table S15), focusing on genes for which functional interpretations can be inferred from genetic studies in mouse and humans (Additional file 1: Tables S12 and 13), as well as genes that have been previously identified as missing in birds and/or that result in a unique avian trait (Additional file 1: Table S6). Since few data are available concerning phenotypes that might result from the loss or disruption of specific genes in lizard (for example, genotype/phenotype studies), inferring predictions based on GO enrichments in Group C is difficult and not a main objective of our study, thus this group of terms was not pursued further.

The set of terms significantly enriched in humans only or in humans/lizard but not in chicken (Group A, Figure 7, yellow; Additional file 1: Table S14A) were found to be associated with a considerable number of genes that have lethal knockout phenotypes in mice and/or severe human disease phenotypes affecting a range of tissues and organs (skin, muscle, bone, nervous system, lungs, immune system, among others; Additional file 1: Table S15A). In contrast, terms exclusively enriched in chicken (Group B_1_) were almost never associated with lethal genes or a severe human disease phenotype. Thus, our functional enrichment analysis was robust enough to detect enrichments of phenotypes/genes that may be exclusively deleterious in mammals. The fact that terms in Group A were not enriched in chicken, suggests that birds somehow compensated for the loss of these vital genes. Indeed, we found some examples within this group where the missing gene has been linked to a change in the expression or post-translation modification of an unrelated gene (for example, *DCN*/*BGN*). In other cases, a close paralog (for example, *ATP6AP1L*/*ATP6AP1*; *SLC6A8L*/*SLC6A8*; Additional file 1: Table S3) or a related family member may have provided compensation.

We found just three terms that were exclusively enriched in chicken (Group B_1_, Figure 7, dark blue; Additional file 1: Table S15B_1_). These are of interest because they indicate functions that may not have been compensated only in birds, and thus could be related to distinctly avian traits. Genes associated with GO terms in Group B_1_ (Additional file 1: Table S15B_1_) were: *NPHS1*, a gene whose loss in humans leads to nephrosis; *NR1H2*, a key regulator of macrophage function; and *KIRREL2*, a novel immunoglobin gene that is expressed chiefly in beta cells of the pancreatic islets. Importantly, terms enriched in human and/or lizard were never associated with this set of genes. Because relatively few functional terms were enriched only in chicken, we postulated that this might be a strong indicator that elements in avian genomes might be providing a functional compensation for gene losses. If true, then we predicted that if we were to analyze a similarly sized set of genes selected at random from the chicken genome, we should observe a greater number of avian enrichments (Group B from Blast2GO analysis). We tested this possibility by conducting a separate functional enrichment analysis on the two randomly selected sets of 274 genes with the same relatively distribution across human chromosomes as the missing gene set. As predicted, we found a nearly two-fold increase in the number of terms in the control set that were significantly enriched in chicken, compared to the missing gene set (that is, Group B genes), thus suggesting that birds may have compensated for the actual gene losses in at least some cases.

Of the GO term enrichments that are shared by all three species (Group B_2_, Figure 7; green; Additional file 1: Table S15B_2_) a subset (31%) are also associated with genes that are either lethal in mice, or related to human genetic diseases or disorders (Additional file 1: Tables S12 and 13 in), including *CEBPE* (congenital granule deficiency), *STXBP2* (hemophagocytic lymphohistiocytosis), and *ATP2B3* (spinocerebellar ataxia). Since these terms are also enriched in chicken, our analysis suggests that the genetic background of birds may not compensate for the missing gene, raising intriguing questions as to how birds might have adapted to and survived the disruption of these vital functions. In other cases within Group B_2_ it appears that the gene loss would have been non-lethal or not associated with a highly deleterious phenotype in mammals, suggesting that the disrupted function might also be tolerated in birds (representative examples are *THPTA*, *CYP2F1*; see also Discussion). Further analysis of this group could reveal associations with other characteristic avian traits, or genetic compensatory mechanisms that were not captured by our functional enrichment analysis.

To further investigate the possible functional impact of the avian missing genes, we next searched for evidence of expressed sequence tag (EST) enrichment in a human gene expression database (Tissue-specific Gene Expression and Regulation (TiGER)) [25]. Not surprisingly, the majority of genes that have known functions (based on inclusion in Additional file 1: Tables S9-13) showed enriched expression in at least one tissue type (Additional file 1: Table S16A). Furthermore, of the 87 genes with no known function, 26 showed enriched expression in at least one tissue type, and several tissues (for example, cervix, eye, spleen, thymus, and small intestine) were found to express several of these genes (Additional file 1: Table S16B). For the remaining 61 genes (Additional file 1: Table S16C), there is currently no information with regards to their tissue-specific expression or functional classification, as these have not yet been studied in detail in any organism. Thus our analysis likely under-represents the functional impact that the set of missing genes may have had for the avian lineage.

### Some missing genes are members of multi-gene families and/or have close paralogs in birds

**Figure 8:**
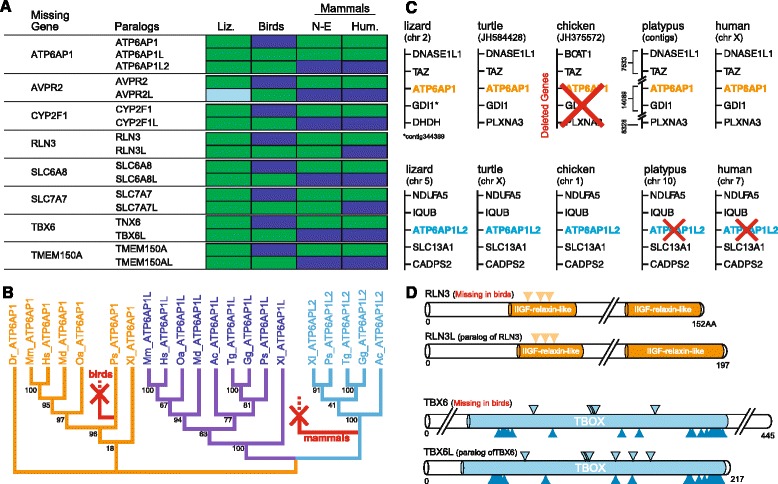
**Missing gene paralogs. (A)** Summary of all newly discovered paralogs of avian missing genes, indicating copies have been independently lost in birds and mammals. Coded cells indicate genes that are present (green), missing (blue), or suspected present (light blue, gene is likely in a genomic gap). Abbreviations: Liz., lizard; N-E, non-eutherian mammal (that is, opossum and/or platypus); Hum., human; pseudo, pseudogene. **(B)** A maximum-likelihood phylogeny of *ATP6AP*-related genes based on protein alignments and rooted to deuterostomes (not shown). Branch numbers indicate bootstrap support; gene losses (in birds and mammals) are in red. Species abbreviations: Dr, *Danio rerio*; Mm, *Mus musculus*; Hs, *Homo sapiens*; Oa, *Ornithorhynchus anatinus*; Md, *Monodelphis domestica*; Tg, *Taeniopygia gutatta*; Gg, *Gallus gallus*; Ps, *Pelodiscus sinensis*; Ac, *Anolis carolinensis*. **(C)** Synteny analysis of *ATP6AP1*/*ATP6AP1L2*. The gray boxes indicate the syntenic block of genes that is deleted in birds (for example, chicken); chromosome or contig number is presented beneath each species name. **(D)** Examples of putative conserved functional domain analysis for paralogous gene pairs. The triangles denote predicted receptor binding sites (orange), a dimerization domain (light blue), and DNA binding sites (dark blue). Further details are in Additional file 1: Table S3. Abbreviations: AA, amino acids.

To identify possible sources of genetic compensation for avian missing genes we next conducted a genome-wide screening for possible avian paralogs, and an orthogroup classification to identify genes that are members of extended multi-gene families; the latter also included a comparative analysis to determine whether orthogroups have undergone expansions in the avian lineage (see Methods for details). A first paralog search using BLAT alignments of the lizard or human ortholog to the chicken genome revealed that eight missing genes have close paralogs in birds (Figure 8; details in Additional file 1: Table S3A). The majority of these are previously uncharacterized in birds, but we found them to be present in lizard and/or in other non-avian vertebrate lineages. These paralog pairs (or triads) are likely to result from duplications in an ancestral tetrapod (not shown). In nearly all cases the novel paralogous gene in a pair (or triad) is absent in humans, although some are present in at least one non-eutherian mammal (Figure 8A). Several of these cases thus illustrate reciprocal gene losses between birds and mammals. Some of the novel paralogs have been misannotated as the missing avian ortholog, but such errors were corrected by our syntenic analyses (Additional file 1: Table S2). As a representative example, *ATP6AP1*, which is associated with GO terms enriched in humans and lizard but not birds, is missing in birds and present in the other vertebrate lineages examined. A previously unidentified paralog (*ATP6AP1L2*) is present in sauropsids (birds and lizard) but missing in mammals, and a different paralog (*ATP6AP1L1*) is present in all extant tetrapods (Figure 8B). The absence of *ATP6AP1* in birds results from an avian syntenic block loss, and is unrelated to the absence of *ATP6AP1L2* in mammals, including non-eutherians (Figure 8C). To address the possibility that paralogs might be able to functionally compensate for the loss of a given ortholog in birds, we analyzed each sequence pair or triad using NCBI’s Conserved Domains Database [26]. In nearly all cases, we found that structural and/or functional domains are conserved across paralogs (examples in Figure 8D; other cases in Additional file 1: Table S3A). With the recent availability of crocodilian genomes, we identified another six cases where the evidence of a novel paralog of a missing avian gene derives from a gene that is in alligator and in bird species, typically not the chicken (Additional file 1: Table S3B); in these cases a predicted model for the novel paralog is not available, thus an analysis of domain conservation was not carried out. Since we directly and exhaustively searched the chicken and other avian genomes by BLAT alignments, we have likely identified the full complement of possible paralogs present in extant birds due to an ancestral duplication of genes in the missing gene list.

For the orthogroup analysis we focused on the 40 genes with deleterious phenotypes and that were associated with term enrichments in our Blast2GO analysis (Additional file 1: Table S15). Using the OrthoMCL database [27], we assigned each gene to a distinct orthogroup (Additional file 1: Table S17), and using OrthoMCL phyletic pattern searches we quantified the number of orthologs present in each orthogroup for a select set of organisms (for example, fish, lizard, platypus, chicken, and humans). These searches revealed that in chicken, 20 orthogroups have one or more members that could have provide compensation for the gene loss. In contrast, the other 20 orthogroups currently have no membership (that is, 0 value, Additional file 1: Table S17), making it unlikely that a related gene family member provided functional compensation. Moreover, we found no evidence that any of the missing gene orthogroups has expanded in chicken compared to lizard or human. We also note that none of the orthogroups previously reported as expanded in birds compared to mammals (see Figure S3 in [4] and Additional file 1: Table S6 and Figure 7 in [5]) are related to the missing genes identified in the present study.

## Discussion

We have presented genomic evidence for the avian loss of 274 protein coding genes located within or in close proximity to conserved syntenic blocks with a clustered localization to discrete chromosomal domains in lizards and humans (human chr19, X; lizard chr2). The majority (86%) of these avian missing genes are present in the crocodilian lineage, and 90% to 95% are present in fish, coelacanth, and frog, suggesting that their loss was largely subsequent to the split of dinosaurs/birds from their archosaur ancestor. These avian missing genes are associated with the physiology of a broad range of organs and systems in mammals, as well as lethality in rodents and severe genetic disorders in humans. Some of them provide plausible explanations for known avian traits, while others were likely compensated by elements of the avian genome, including novel paralogs. As discussed below, these findings have important implications for understanding several aspects of avian physiology and the evolution of avian traits and adaptations. They are also potentially important for developing novel animal models for human disease, and could be of relevance to the poultry industry.

### Evidence supporting the loss of protein coding genes in birds

We have high confidence that the genes on our final curated set are absent in birds. Our approach was conservative, focusing on genes that are part of, or closely related to syntenic deletion blocks within discrete chromosomal domains. We also excluded genes for which syntenic verification was not possible. While this approach likely underestimates the full extent of gene losses in birds, it effectively minimizes the chance that genes on our final set might be present, but undetected in the avian genomes analyzed. Our approach included comprehensive and manual searches and synteny verification in the most fully sequenced and annotated avian genomes (chicken, turkey, zebra finch, medium ground finch, and budgerigar), tBLASTn searches of the complete set of available whole genome shotgun contigs in NCBI (60) followed by manual verification of significant hits, and BLAST searches of avian EST/mRNA collections. Moreover, given the large average size of the missing syntenic blocks in lizards and humans, the cumulative size of these missing blocks, the high coverage of the latest chicken genome assembly based on combined Sanger, 454, and BAC sequences and improved assembling algorithms (18X; galGal4.0) [28], and the fact that the various other avian assemblies are largely based on yet a different sequencing technology (Illumina), it is extremely unlikely that the non-detection of the missing sequences in birds is due to lack of sequence coverage or assembly problems. Providing independent validation, previous studies that utilized independent database searches and/or molecular verification techniques (for example, PCR amplification, southern blot analysis, molecular cloning, or purification of protein or corresponding biological activity from avian tissues) have concluded that several genes on our missing gene list are absent in different bird species. We also note that our combined efforts resulted in a much better and exhaustive curation of avian genomes. Lastly, compared to other birds, the chicken genomic and transcriptome sequences lack yet a further subset of genes, which we suggest may represent losses specific to chicken or to galliformes, but since we were searching for genes whose absence is a general feature of birds, these were excluded from our final set.

A possible concern is that our searches of avian genomes might have missed genes that are rapidly evolving and have highly divergent sequences across vertebrate lineages. In addition, recent studies suggest that short genes may be more rapidly evolving, which in some cases can lead to errors in the identification of orthologs in large phylogenies (for example, [29]). Indeed, sequences from some avian missing genes do not cross align, and their predicted proteins show <50% identities between lizard and humans. However, we believe that these concerns are minimized for several reasons. First, the low conservation genes represent only a fraction of the avian missing genes; a much higher percentage of these genes were found to have surprisingly high conservation across non-avian organisms. Second, our analysis demonstrates that the missing genes are not disproportionately enriched in small genes (that is, <500 bp), when compared to the full complement of genes that are present in birds. Third, we find no evidence within the missing gene set for a correlation (either positive or negative) between gene length and the degree to which the gene has diverged across non-avian organisms. Finally, we note that: (1) even low conservation genes can be found in cross-species BLAT/BLAST alignments when they are present in an avian genome; (2) in their vast majority, low conservation genes from our list could easily be found in cross-species alignments with crocodilian genomes, which are phylogenetically closer to birds than other non-avian sauropsids; (3) we used probes from multiple species, including from crocodilians when available, as queries in our searches for low conservation genes in avian genomes. It is thus highly unlikely that our results can be explained by lack of detection of orthologous sequences in avian genome databases due to low sequence conservation.

We compared our findings to the recently completed analysis that used BLAST alignments of human protein coding sequences to a set of 48 avian and five non-avian reptile genomes. That study identified 640 genes as missing or representing likely pseudogenes in modern birds ([7]; Additional file 1: Table S8). Surprisingly, the lists from the two studies have a relatively small overlap (91 genes), constituting approximately 33% of the genes we are reporting as missing in birds (Additional file 1: Table S1; genes discovered by both studies indicated by a ‘^’). While these studies partially corroborate each other, it is important to highlight the differences, which largely relate to the different approaches used. Here, we specifically screened for missing genes that are in highly conserved syntenic blocks in human and lizard, not just in sauropsids. While our initial search revealed >1,500 candidate missing genes, approximately 1,000 are either singletons or pairs in small unassembled segments (<80 kb) of the lizard genome, thus they were not investigated further due to the concern that they may be present in unsequenced portions of avian genomes. We also focused on the subset of protein coding genes of the human genome (12,000 out of 21,000) that have 1-to-1 orthologs in lizards. This was necessary because relying on 1-to-many or many-to-many orthologies complicates substantially the task of syntenic verification and often leads to incorrect ortholog identification. We also used highly stringent criteria to confirm the validity of the missing genes, including comprehensive and manual searches of high quality avian genomes, genome trace archives, and EST/mRNA collections. This effort revealed that a large subset of the initial 538 candidate missing genes is actually present in birds (Table 1). Although limited manual curation was conducted in the Zhang *et al*. study [19], it was not done for all genomes given the large number of species examined. Indeed, we have found evidence that 35 of the genes reported as missing in that study are likely present in some birds. We note though that all of the species we interrogated were included in the Zhang *et al*. study, and that some of these 35 genes may only be partial or a pseudogene. In sum, the present study provides a well-curated analysis of missing avian genes that is largely complementary to the findings of Zhang *et al*. Together, these studies may come close to identifying the full complement of genes that were lost in an avian lineage ancestor. As further higher quality sauropsid genomes become available, it should become possible to further refine the full extent and evolutionary history of gene losses specific to the avian lineage.

### Evidence for syntenic gene loss

The syntenic blocks of missing genes in birds are mostly localized to discrete domains in lizard and human chromosomes. The flanking genes to most of these missing gene blocks in humans are either present in different chromosomes or in very different positions of the same chromosomes in birds. This observation hints that chromosomal rearrangements involving syntenic blocks may have been a main contributor to the loss of protein coding genes that we have discovered in birds, as opposed to the independent deletion of individual genes in an avian ancestor. Interestingly, human chromosome 19, a relatively short but highly gene dense chromosome where rearrangements and segmental duplications are frequent [30], is the major location for the avian missing gene blocks. This is again consistent with view that the avian losses were likely derived from extensive rearrangements of chromosomal segments in an ancestral species. In lizard, the majority of avian missing genes and corresponding blocks localize to small contigs that are unplaced in the current assembly. In fact, many of these unplaced contigs correspond to entire avian missing blocks, possibly resulting in an underestimate of the conserved deletion block size. We thus suspect that the actual size of the avian missing blocks, representing ‘chunks’ of an ancestral genome, may turn out to be even larger once a better lizard assembly becomes available.

The set of avian missing genes is highly conserved throughout the vertebrate phylogeny, approximately 95% of them being present in sarcopterygians, and approximately 90% in teleosts. Thus, the majority of the missing avian genes were likely present in a sarcopterygian ancestor, and lost sometime after the split of dinosaurs and birds from their common archosaur ancestor. Moreover, only a small subset of these avian missing genes were lost in a non-amniote vertebrate lineage, where such losses were dispersed throughout the genome, and not in syntenic blocks. To our knowledge there are no reports of comparable syntenic gene losses in other vertebrates. For example, although the teleosts are known to have undergone whole genome duplication (WGD) [31,32], and subsequently lost a significant number of protein coding genes [33], we have found no reports indicating that these losses were syntenic; instead, they appear to have occurred in a distributed manner throughout the genome. In fact, in a recent comparison between representative species of different teleost lineages (tetraodon and zebra fish) [34], the losses of various paralogs were shown to be largely reciprocal, occurring in an interspersed and distributed manner in paralogous chromosomes instead of in syntenic blocks in each lineage. Thus, the loss of a substantial number of genes in conserved syntenic blocks that are localized to discrete segments of specific chromosomes (Figures 3 and 4) appears to be a uniquely avian phenomenon among vertebrates.

### Refining the origins of the avian gene loss

We found evidence for the presence of a large proportion (86%) of the avian missing genes in crocodilians, an observation further supported by our previous detection of one of these genes (*SYN1*) in crocodile through PCR amplification [4]. Thus a substantial number of avian missing genes were lost after the split of dinosaurs/birds from crocodilians (Figure 1). This is also consistent with the suggestion that the genomes of sauropod dinosaurs, which were closer to therapods and therefore to extant birds, were also relatively compact, while those of ornithischian dinosaurs, which were closer relatives of crocodilians, were larger [15]. We note, however, that a detailed syntenic analysis of all significant hits to crocodilian genomes will be required in order to more definitively establish the orthology of these crocodilian loci. We also note that, in spite of a reasonably good coverage in crocodilian genomes (>70X), as attested by a large percentage (91%) of BLAT-alignment hits from the positive control gene set, several genes on our avian missing gene set were only found in one or the other of the two crocodilian species examined. While some genes may have been differentially lost across crocodilian species, or significantly diverged between lizard and crocodiles, it seems more likely that the current crocodilian genome assemblies are incomplete. Thus, the percentage of avian missing genes we detected in alligator/crocodile is likely an underestimate, and an even larger subset of these genes may actually be present in crocodilians. Alternatively, a small but significant subset of the avian missing genes we discovered may also be absent in crocodilians, and thus have resulted from a loss in an ancestral archosaur. More definitive answers to these possibilities await further completion and annotation of crocodilian genomes. Interestingly, except for two genes, all the 274 genes missing in modern neognaths - all living birds with the exception of the paleognaths (that is, tinamous and ratites), are also missing in ostrich, a basal ratite. Thus, practically the entire set of avian missing genes was lost prior to the split between neognaths and paleognaths.

### Functional implications of avian missing genes

How did birds adapt to and survive the loss of such a large number of protein coding genes, many of which associated with vital functions and pathways in other tetrapods? For the subset of genes linked to tissues, organs, or traits that are absent in extant birds (for example, teeth, hair, mammary glands, and placenta, their losses may not have been deleterious, and in some cases may have even co-evolved with the trait loss. For several other avian missing genes we found novel, previously undescribed paralogs. These paralogous pairs or triads are for the most part present in lizard and thus were likely present in ancestral amniotes, but the mammalian vs. avian lineages have retained different members. Most of these paralogs have nearly identical functional domains as the avian missing orthologs, and thus may have provided compensation if expressed in the correct target organ. For example, *SLC6A8*, which is linked to a creatine deficiency syndrome that causes mental retardation, severe speech delay, and seizures, and *SLC7A7*, which causes lysinuric protein intolerance are both missing in birds (OMIM), but have closely related paralogs that could provide compensation. In contrast, we have found that birds lack *AVPR2*, the kidney antidiuretic hormone receptor, whose loss in humans causes a genetic form of diabetes insipidus [35]. Although this loss could be functionally compensated by a close paralog (*AVPR2L*), which is missing in other lineages including mammals, birds possess a lower capacity to concentrate urine in response to blood hyper-osmolarity compared to mammals [36]. Thus, while AVPR2L may have provided some compensation for a highly detrimental gene loss, this compensation may be only partial.

A much larger number of genes are associated with vital functions involving a range of important organ systems and pathways, and their loss would have been highly deleterious if occurring in other organisms. Since little is known about the function of most of these genes, particularly in the context of lizard and avian genomes, we decided to conduct a Blast2GO enrichment analysis. The goal was to gain a better understanding of some of the possible implications of gene loss in birds. According to our comparative Blast2GO gene classification, and considering the impact of gene loss in different genomic contexts, a considerable set of missing genes have GO annotations enriched in humans/lizard but not in birds, pointing to cases where the loss seems to have been compensated in the context of avian genomes, and thus likely well tolerated by birds. Several genes in this group are part of families or orthogroups, some with several members that may have provided compensation for specific losses. As an example, *BCAT2* is absent in birds, but BCAT-related activity has been detected in avian tissues like muscle and liver [37], helping prevent deleterious hyperaminoacidemias in birds. This activity likely derives from a compensatory expanded expression of *BCAT1*, the other gene in this orthogroup, consisting of a cytosolic isoform, which in mammals has predominantly brain and placental expression [37]. Also consistent with this possibility, some avian missing genes are only lethal in mice when combined with a knockout of a related family member. Other genes in this group are not part of multi-gene family members but compensatory changes have been reported in the expression or biochemical properties of proteins from related but different families (for example, *BGN*/*DCN*). In other cases, however, a possible avian compensatory mechanism and/or functional impact for the avian gene loss is unknown, including cases of severe disease or lethal phenotypes when the genes are deleted in other organisms, such as *ABCD1* and *PRX* (central and peripheral demyelinating diseases), *FGD1* (affecting bone growth), and *FTSJ1* and *SYP* (X-liked mental retardation). Of particular interest are human disease-causing genes that are lethal in mice, which would create considerable difficulties in developing appropriate rodent models for their study. Future in-depth analysis of other genes in Group A will likely reveal further compensatory mechanisms that allowed birds to adapt to and tolerate their losses. This in turn could lead to basic insights into the pathophysiology of human genetic diseases, and potentially to novel avenues for the treatment and/or cure of these disorders.

The loss of the set of potentially deleterious missing genes associated with enriched GO terms in birds only was likely compensated in other vertebrates but apparently not in birds. These genes possibly reflect traits that are specific to birds. As an intriguing example, *NPHS1*, which results in kidney nephrosis and disruption of the glomerular filtration barrier when functionally knocked out in humans, and *KIRREL2*, which is expressed in kidney and encodes the slit diaphragm protein Neph2/filtrin [38,39], are both missing in birds, possibly leading to a reduced control of glomerular filtration rates compared to mammals. These cases would help explain the lower capacity of birds to concentrate urine under a hyperosmotic challenge, and could relate to the emergence of the birds’ ability to regulate water/electrolyte balance by modulating water release from red blood cells [40]. Combined with the avian lack of *AVPR2*, the kidney appears to be a major target system of avian missing genes.

For other genes whose absence is potentially highly deleterious, the related GO term enrichments are shared by birds, lizards, and humans, suggesting that there are no apparent compensations in any of these genomic contexts. Indeed, most genes in this set belong to very small gene families and/or orthogroups with only one or no additional members. This again raises intriguing questions in terms of possible compensatory adaptations. Some cases are discussed in the next paragraphs.

More than 20 missing genes are involved in erythropoiesis, the process of red blood cell production in the bone marrow, which could have important implications for the ability of birds to respond to hypoxic conditions. Interestingly, the products of two avian missing genes (*EGLN2* and *HIF3A*) are known to suppress the cellular response to hypoxia [41-43]. A possible prediction is that hypoxia-responsive genes may be more highly expressed in avian tissues compared to other organisms, or be more rapidly elevated under hypoxic conditions. This in turn could potentially provide functional compensation for the absence of several genes involved erythropoiesis.

Several other missing genes in this Group B_2_ appear to be tightly correlated with specific avian molecular or biochemical traits that are also worth mentioning. For example, the loss of *PTGIR* provides a likely explanation for the known and puzzling lack of responsiveness of chicken platelets to prostacyclin [44], the most potent anti-aggregation factor in mammals, indicating that other prostaglandins are likely involved in hemostatic function regulation in birds. Avian brain tissue is also known to have high levels of ThTP (thiamine-triphosphate, the triphosphate form of Vitamin B1, or thiamine) than ThDP (thiamine-diphosphate) compared to other tissues and organisms [45]. This fact can be explained by the loss of *THTPA*, a mammalian brain-expressed enzyme that converts of ThTP to TDP. The loss of *CYP2F1*, a lung-expressed cytochrome *P450* related gene involved in the bioactivation of pulmonary-selective toxicants [46], explains the avian lack of the lung enzymatic activity involved in generating the endotoxin 3-methylindole [47]. This in turn would explain the avian insensitivity to repellents such as naphthalene, also a substrate for this enzyme [48]. Even though we have discovered a paralog for this missing gene, it is unclear whether it is present in the lung, where its expression would be needed to compensate for the gene loss.

## Conclusions

In sum, our findings provide a more accurate understanding of the avian genetic makeup as well as novel insights into the evolutionary origins of gene losses affecting the avian lineage. We also highlight a number of examples wherein birds constitute natural knockouts for genes that in other organisms are known to play fundamental metabolic or physiological roles, or are associated with severe disease phenotypes and genetic disorders. It is also noteworthy that the function of numerous avian missing genes described here relate to areas of biomedical research to which birds have made substantial contributions as model organisms, including development, immune system function, oncogenesis, and brain and behavior, to name a few. It will be important to assess the impact that avian gene deletions might have for these fields of research. Our studies have also identified a number of gene deletions as well as possible compensatory adaptations that have important implications for understanding basic aspects of avian physiology, and could be of potential relevance for improving commercial poultry strains.

## Materials and methods

### Identification of syntenic blocks of missing genes in birds

In order to identify gene losses that occurred in the avian lineage, we performed a comparative genomics analysis in humans (*Homo sapiens*); a lizard (green anole; *Anolis carolinensis*) representing a non-avian sauropsid; two galliformes (chicken; *Gallus gallus*; turkey, *Meleagris gallopavo*) representing a basal avian order; and an oscine passeriform (zebra finch; *Taeniopygia guttata*). These representative species currently have the most well-assembled and annotated genomes within their respective taxonomic groups. To extend this initial analysis we also examined two additional non-avian sauropsids, the painted turtle (*Chrysemys picta bellii*) and the American alligator (*Alligator mississippiensis*) to further identify human/sauropsid orthologs. Our rationale was that genes that are present in non-avian sauropsids and mammals, but absent in these representative species from distantly related avian groups likely correspond to gene losses that are characteristic of the avian lineage, rather than reflecting genomic features that are specific to lizards or to specific avian species. For consistency we use human gene naming conventions (HGNC) [49] whenever possible throughout this paper.

To identify genes missing in avian genomes we first retrieved from Ensembl BioMart the full list of lizard Ensembl gene models (Broad AnoCar2.0/anoCar2) with their respective chromosomal locations, and identified a subset that had 1-yo-1 orthologs (including apparent 1-to-1 orthologs) in humans (GRCh37.p10/hg19). Within this 1-to-1 ortholog set we next searched for genes with 1-to1 orthologs in chicken (ICGSC Gallus_gallus-4.0/Galgal4) and/or zebra finch (WUGSC 3.2.4/taeGut1). Among these were 1-to-1 orthologs in lizard and humans that have no corresponding Ensembl orthologs in either chicken or zebra finch, and thus are possibly missing in birds. We noticed that a subset of the presumed missing genes in birds have clustered chromosomal locations in lizard and humans, suggesting an organization into syntenic blocks. To further investigate this possibility, we sorted all the identified 1-to-1 orthologs in lizard and humans side by side with the subset of identified orthologs in chicken and zebra finch, initially based on chromosomal location in lizard, and confirmed that a large number of missing genes in birds are clustered into syntenic blocks in both lizard and humans. We next manually scanned the entire list and identified and numbered all syntenic blocks of genes that are present in lizard and human but missing in birds, and that also meet either of the following criteria: (1) the block is at least 80,000 bp in size from the start of the first gene to the end of the last gene in the block, based on Ensembl model coordinates in lizard; or (2) the block contains at least three adjacent genes. In some cases we used the assembled painted turtle genome (v3.0.1/chrPic1) to identify/confirm the syntenic gene order within missing blocks that are located in poorly assembled regions of the lizard genome. The identified blocks are represented in dark orange on Additional file 1: Table S1A. We also identified additional blocks of missing genes consisting of singlet or doublets that were at least 80,000 bp in size, or of doublets whose average size was approximately 34,000 bp (shaded in medium and light orange, respectively, on Additional file 1: Table S1A). This allowed us to also include pairs of missing genes that are very large. After numbering the syntenic missing blocks in lizard, we realigned the entire list based on chromosomal location of orthologs in humans, and again eliminated any genes that did not meet the inclusion criteria above. This was necessary to identify any differences in chromosomal alignments between lizard and humans reflecting chromosomal rearrangements and that could affect the organization of the syntenic blocks we detected. Overall, this approach allowed us to identify highly conserved blocks of genes that have nearly identical syntenic organization in lizard/turtle and humans but that are missing in birds. We also noticed several cases where presumed avian orthologs (based on the existence of an Ensembl model in at least one avian species) disrupted an apparently larger missing syntenic group, even though the large majority of these avian models were themselves unplaced in the corresponding assemblies (Additional file 1: Table S18). We took a conservative approach and interpreted these Ensembl models as evidence of the presence of these genes (even if only partial) in birds, although a syntenic confirmation of their identity was not possible. Further investigation of these gene models that are putatively present in avian genomes will be an important future goal.

### Curation and annotation efforts

To refine the syntenic analysis, we manually examined the corresponding genomic regions in all four species above (plus turtle and American alligator as needed) in order to verify the correctness of the predicted syntenic blocks, including the position and orientation of orthologous genes. While performing this curation, we found that the syntenic blocks often contained further genes that were initially not included due to the lack of a predictive Ensembl model in lizard. In such cases, we retrieved the predicted nucleotide and/or protein sequences from human, and BLAT aligned them to the lizard genome using the UCSC web browser to confirm the gene is present and in the correct syntenic position (Additional file 1: Tables S1A and B, ‘no model’ cases). In several additional cases we noted that the Ensembl models in lizard were not included in the missing syntenic blocks because they were not annotated as 1-to-1 orthologs to the corresponding Ensembl models in humans. In most such cases we were able to identify the correct orthology by BLAT alignments and synteny analysis using the human orthologs as queries (Additional file 1: Tables S1A and B, Lizard Ensembl Gene ID Column, Ensembl models indicated with an ‘†’).

To address possible errors in the orthology annotations in Ensembl, we next examined whether Ensembl had chicken or zebra finch entries for any of our predicted missing genes. Because a gene prediction set from any given database is likely to be incomplete, we also examined whether there were entries that matched the name or gene description of any of our predicted missing genes in other existing chicken and zebra finch databases (Entrez Gene, UniGene, and RefSeqs). We also examined a recent set of chicken gene predictions by Ensembl (release e71), which incorporates more extensive transcriptome data, as well as the gene predictions from all the databases above for three other avian genomes available in NCBI: turkey (Turkey Genome Consortium; Turkey_2.01/melGal1), medium ground finch (Beijing Genomics Institute; GeoFor_1.0/geoFor1), and budgerigar (WUSTL and E. Jarvis; v6.3/melUnd1). We also searched the NCBI’s avian nucleotide databases for any evidence of cloned mRNAs in birds that might be annotated as a gene on our missing set. For all the searches above, we manually examined all entries that matched a gene on our missing gene list. Specifically, we systematically BLAT aligned all the reported sequences to lizard, turtle and other genomes and/or BLAST searched the entire NCBI’s nucleotide or protein databases and verified the percent identity and synteny of significant hits. Any confirmed positive hits were excluded from our list of missing genes; the evidence for their existence in avian genomes is presented in Additional file 1: Table S4. All other hits, typically consisting of hits to related gene family members and paralogs, were considered false positives; the evidence for this curation/annotation effort is presented in Additional file 1: Tables S2 and S3. In some cases positive identity could not be definitely established as the hits were short or to unplaced contigs, preventing a syntenic analysis. However, we took a conservative approach and removed such cases from our missing gene list, since they provided suggestive evidence of the presence of the gene in birds. In several cases this approach resulted in some of the final syntenic blocks being shorter than in the initial analysis, and in some genes being moved to the category of missing genes that do not directly belong to a missing syntenic block (Additional file 1: Table S1B).

### BLAT/BLAST searches for missing genes in birds

To further confirm that the genes in the identified syntenic blocks are indeed missing in avian genomes, we next conducted a series of BLAT/BLAST searches for genes on our missing list using updated assemblies of the chicken and zebra finch genomes. For all BLAT searches, we used a local BLAT server [50] and house scripts with parameters set to be highly permissive of divergent and incomplete sequence alignments, accepting and manually curating any hits that had an alignment score >50. We note that this cutoff was first established based on the manual curation of hits of lower scores for more than 100 missing genes; in every case, the low scoring hits were to loci not associated with the missing gene, and typically consisted of just a short segment of a single exon from a related gene family member. After establishing this criterion, we BLAT-aligned the complete set of predicted coding DNA sequences (CDSs) from the lizard Ensembl models of missing genes to the assembled genomes of chicken and zebra finch. This procedure allowed us to identify genes that might be present in avian genomes but that were not identified by Ensembl or by other predictive algorithms displayed on UCSC’s or NCBI’s genome browsers. We noticed that in some cases a lizard gene model itself is missing, usually because the gene sequence is truncated due to a gap in the lizard genome assembly. In such cases we conducted the BLAT-alignment to avian genomes using the CDSs from the human Ensembl genes. We note that we used the most recent version of the chicken genome (galGal4), and an improved version of the zebra finch genome (Mello and Warren, unpublished data) in which additional Illumina sequence data were used to partially fill in the gaps present in the zebra finch genome assembly currently available in NCBI. To address the possibility that some of the genes on our list might be present in unassembled portions of the best-covered avian genome, we also conducted mega-BLAST searches of the individual genome sequencing reads for chicken and zebra finch [51], and an Illumina SOAP *de novo* chicken genome assembly (Warren lab). For all BLAT and BLAST searches, we manually verified all significant hits. The vast majority of hits were to well-assembled regions of the genomes, which allowed us an accurate assessment of orthology through synteny. We verified that the hits were typically to related gene family members or paralogs, which therefore were considered false positives (Additional file 1: Table S5). With regards to hits to segments that are unplaced in the assembly, in some cases the unplaced segments were large enough to allow direct verification of gene orthology based on synteny. In the other cases we retrieved the target sequences in chicken and BLAT aligned them to the genomes of several organisms (including lizard, turtle, frog, and human) to confirm gene identity by sequence similarity and synteny. In all cases we also performed BLAT alignments to other avian genomes since we noticed that other species, in particular the budgerigar and medium ground finch, have better coverage of specific genomic regions than chicken or zebra finch. Finally, to address the possibility that some of the missing genes might only be present as cloned mRNAs/ESTs, and not represented in any current avian genome assemblies, we conducted a separate series of nucleotide (BLASTn) and protein (tBLASTn) searches of the available chicken EST (for example, BBSRC, Univ. Delaware Chick EST) and avian core nucleotide and protein databases (for example, NCBI). All BLAST searches used conservative parameters (Block Substitution Matrix 45) for highly divergent sequences. Any confirmed positive hits for the BLAT/BLAST searches were eliminated from our avian missing gene list (Additional file 1: Table S4B). In several cases this conservative approach resulted in the shortening of some further syntenic blocks that were initially larger, and in several further genes being moved to the category of missing genes that do not directly belong to a missing syntenic block (Additional file 1: Table S1B). Importantly, for all BLAT searches of avian databases conducted using the lizard models as queries, we also included a parallel set of 500 randomly selected protein coding genes in lizard that have 1-to-1 orthologs in humans, chicken, and zebra finch as a positive control to ensure the effectiveness of the search algorithm and the adequacy of using lizard models for cross-BLAT searches in birds.

### Expanded curation and alignment searches of avian genomes

A large number (45) of avian genomes beyond those used in our initial analyses have been recently completed in the context of the Avian Phylogenomics Consortium (Additional file 1: Table S1 in [7]; datasets available at [52]), or have been made publically available by various research groups (n = 12; Puerto Rican parrot, *Amazona vittata*; Golden Eagle, *Aquila chrysaetos*; Scarlet macaw, *Ara macao*; Northern bobwhite, *Colinus virginianus*; Hooded crow, *Corvus cornix*; Japanese quail, *Coturnix japonica*; Saker falcon, *Falco cherrug*; Collared flycatcher, *Ficedula albicollis*; Black grouse, *Lyurus tetrix*; Tibetan tit, *Pseudopodoces humilis*; Canary, *Serinus canaria*; White-throated sparrow *Zonotrichia albicollis*); a subset of these have RefSeq annotations. These resources have allowed us to greatly expand our curation and alignment searches of avian genomes as described in the previous sections, to include a broader range of species with much more extensive phylogenetic coverage, including all the main branches of the avian tree of life [8]. To search these genomes for any evidence of the avian missing genes in our curated candidate set, we first examined RefSeq annotations. All entries with the same gene names as our candidate missing genes, or with the same main key terms in their gene descriptions were examined for orthology, including reciprocal cross-alignments with non-avian probes and synteny verification when possible. We also performed tBLASTn searches of the corresponding WGS databases of all these genomes. To address the possibility some of the candidate missing genes might have divergent sequences from their non-avian orthologs so that we might have missed them in our previous searches due to low conservation, we took the following additional steps for selecting query sequences for our searches: (1) examined the candidate missing genes for their BLAT scores in cross-species alignments and the % identities of their Ensembl protein sequences (lizard vs. human comparisons); (2) classified them into high vs. low conservation sub-sets, based on the verified BLAT alignment scores and % identities; (3) verified the presence of orthologs in crocodilians (alligator) for the low conservation gene subset; (4) utilized probes from multiple species the low conservation candidate missing gene subset, including alligator when available, as queries in the tBLASTn search of avian WGS databases. As in the preceding sections, all significant hits were manually verified by cross-reciprocal alignment tests and synteny verification when the avian hit presented sufficient flanking sequence.

To compare the relative distributions of genes according to size, and rule out the possibility that the missing gene set was particularly enriched in short genes, we constructed frequency distribution plots of protein coding sequence length (CDS) for the missing gene set and the complete set of lizard genes present in birds (Additional file 3: Figure S2A). Distributions were normalized by log-transformation and compared using a two-tailed ANOVA (α = 0.05).

To test whether gene size was correlated with protein coding sequence divergence we retrieved (from Ensembl Biomart) the amino acid percent identities (% AA; lizard vs. human orthologs) for the full set of avian missing genes. Genes lacking a clear 1-to-1 orthology, or that did not have an Ensembl model prediction, as was true for several lizard genes, were excluded from further analysis. We then plotted each gene’s CDS length as a function of its % AA identity, but found no significant correlation between these two variables.

### Analysis of chromosomal location of avian missing genes

To test whether the distribution of the avian missing gene blocks was significantly different from a uniform random distribution (as was apparent from the frequency distributions presented in Figure 3), we conducted a contingency table analysis using a Chi-squared test for independence. We reasoned that if the deletion events had occurred randomly and uniformly across all chromosomes, then the larger chromosomes should contain the largest proportion of deletions. To address this, for each chromosome we first calculated the number of deletion blocks that would be expected based on a random and uniform assignment of all 52 missing blocks based on chromosome size. We then applied a pair-wise Chi-squared test for independence (α = 0.05; Prism; Graphpad) to determine whether the observed distribution of deletions was significantly different from the expected random distribution.

To test whether the distribution of individual gene losses differed significantly from a random distribution, we compared the distribution of all 274 genes missing in birds, including singlets, to an equivalent distribution constructed by taking the average chromosomal positions of a set of 274 genes, selected randomly 10 times from the entire collection of genes in the avian genome. We applied a pair-wise Chi-squared test for independence (α = 0.05; Prism) to determine whether the observed distribution of gene deletions was significantly different from the average randomly selected distribution.

To compare the sizes of the missing avian blocks in lizard vs. human chromosomes, we first calculated the size of each corresponding syntenic block (in Mb) by subtracting the start position of the block from the end position based on the Ensembl gene model coordinates. In cases where the lizard genome was poorly assembled, or contained many gaps, we substituted the coordinate calculations based on the turtle assembly. We then compared the distributions of the blocks for all chromosomes, as well as separately for some individual chromosomes, by performing a pair-wise comparison of individual blocks by using a non-parametric Wilcoxon matched-pairs signed-ranks test (α = 0.05; Prism). To calculate the total size of the avian missing blocks in humans and lizard, we added together the sizes (in Mb) of each of the 52 individual blocks.

### Searches for avian missing genes in crocodilians and non-amniotes

In order to refine the evolutionary history of the avian gene loss, we BLAT-aligned our curated avian missing gene set using lizard and human Ensembl CDSs to two recently available, high-coverage crocodilian genomes, the American alligator (*Alligator mississippiensis*) and the saltwater crocodile (*Crocodylus porosus* [11,12]), using our local BLAT server and sensitized parameters as described above for avian genome searches. Because these crocodilian genomes are currently not fully assembled or annotated, and the scaffolds are not long enough for a full-scale syntenic analysis, we determined gene presence or absence by comparing the total number of hits in alligator and crocodile to those in chicken, using alignment score and percent identity to separate hits to the orthologous lizard and human genes from hits to related gene family members that are also present in chicken. Genes were considered present in crocodilians if they fell within the following criteria: (1) the gene had a significant hit to either alligator or crocodile but not to birds; or (2) the gene had hits to both crocodilians and birds, but at least one of the crocodilian hits was of substantially higher score and percent identity that those to birds, and the latter were shown to be hits to related gene family members or paralogs (Additional file 1: Tables S3 and S5).

To further refine the evolutionary history of the avian missing genes we conducted a separate orthology analysis across a set of representative vertebrate species, including lamprey (*Petromyzon marinus*), two teleosts (*Danio rerio*, *Takifugu rubripes*), coelacanth (*Latimeria chalumnae*), and frog (*Xenopus laevis*). For each of these species, we used Ensembl’s Biomart [53] to retrieve a complete set of orthologs for each avian missing gene. To determine the extent to which the missing genes were present in the various vertebrate lineages, we sorted the entire set of orthologs present in frog, and identified specific cases where no ortholog was predicted. We then confirmed the presence (or absence) of orthologs in each of the other vertebrate lineages. We repeated this analysis for each species in order to identify cases where a gene was: (1) present in coelacanth and frog, but not fish, indicating gene likely appeared in the sarcopterygian lineage; or (2) present in coelacanth, frog, and fish, indicating the gene was present in an ancestral teleost. Next, we searched for cases where avian missing genes were specifically absent in frog, coelacanth, or fish, but present in the other species. All putative losses in fish were confirmed by directly searching for evidence of an ortholog in lamprey. Finally, for each species, and each set of gene losses, we determined the relative human and lizard chromosomal positions, and searched for cases where the losses were syntenic.

### Identification and supportive evidence for paralogous gene pairs

In some cases a lizard (or human) mRNA and/or protein coding model used as a query had a particularly high BLAT alignment and identity score (>90%) to one or more loci in zebra finch and/or chicken whose synteny did not match the synteny of the query gene or of a related gene family member in lizard. Such hits presented a reasonable likelihood that the avian locus might represent a previously unidentified paralog. To address this possibility, we used a comparative analysis of synteny to fully annotate the avian locus by searching for a corresponding locus in lizard and other non-avian vertebrates. First, we determined the synteny of the avian region by walking the chromosome (or contig) and documenting the order of genes immediately flanking the locus identified by the BLAT hit. In cases where the BLAT hit in chicken and zebra finch was to a short unplaced segment without clear synteny, or to a disrupted region that contained multiple genomic gaps, we relied on separate BLAT and synteny analyses in budgerigar and/or medium ground finch. We next determined whether the lizard genome might contain one or more closely related paralogs by BLAT-aligning the lizard query back to the lizard genome. In positive cases, we next determined the synteny of the resulting hits in lizard by examining the flanking genes of the high scoring hits. This analysis led to the identification of novel (most unannotated) paralogs in lizard. We next compared the synteny of the high scoring hit in avian lineages with the syntenies of the multiple hits in lizard and found cases where the syntenies in birds matched those of the newly found paralogs in lizard. To further characterize these cases of paralogy, we performed a more comprehensive synteny and phylogenetic analysis based on the presence or absence of the paralogous gene pair across a select set of vertebrate genomes, including non-eutherian mammals (that is, opossum and platypus), and a representative eutherian mammal (that is, human). A summary of the results of this analysis are presented in Figure 8A, with details on Additional file 1: Table S3. A representative example of the synteny analysis is presented in Figure 8C. To reconstruct the evolutionary history of these paralogs we retrieved the corresponding protein coding sequences for each, performing multiple protein sequence alignment using PRANK [54] with stringent substitution scoring and otherwise default parameters via the WebPrank server [55]. These alignments were used to construct maximum likelihood phylogenetic trees using PhyML [56], using the approximate Likelihood-Ratio Test to compute branch support. An example of this analysis is presented in Figure 8B. Lastly, we analyzed the sequences in each pair of paralogs using NCBI’s Conserved Domains Database [26] and performed a side-by-side visual comparison in order to identify conserved domains as well as DNA and protein binding sites that are related to the established function of the gene (examples in Figure 8D, details in Additional file 1: Table S3).

In addition to the above searches, we also used orthogroup classification (via OrthoMCL; [27]) to identify whether the avian missing genes would have been members of a multiple-gene orthogroup, and/or whether a possible paralog might be present in birds, but not lizard or humans. We first used OrthoMCL to assign the missing gene set (439 genes using lizard/human protein sequences), and 13,101 extant chicken genes (Ensembl; e71) to an OrthoMCL group. We note that every missing gene was successfully assigned to an Orthogroup with the exception of *FFAR1*. We then searched for cases where a missing gene was present in an OrthoMCL group that contained additional members. We were able to confirm that most of the paralogs we found using genome-wide screens (see above) were present in the same orthogroup as the missing gene, providing an independent confirmation of the approach. For cases where one or more member of a missing gene orthogroup was found we then retrieve the corresponding gene names, since such genes might provide possible functional compensation for the missing genes.

### Bioinformatics and functional classification

In an attempt to categorize the identified missing genes, we subjected the entire curated set (Additional file 1: Table S1) to Ingenuity Pathway Analysis (IPA; Qiagen, Inc.). The complete set of 274 genes (as HGNC symbols) was uploaded and contrasted against the Ingenuity Knowledge Base Reference Set (Genes Only) to identify pathway enrichments. Only relationships where confidence = ‘Experimentally observed’ were included in the final analysis. This analysis revealed broad categories that were enriched in genes related to diseases and disorders, molecular and cellular functions, and/or physiological system development and function (Table 2A). In addition, this analysis revealed specific diseases states (Additional file 1: Table S9), as well as canonical pathways (Table 2B) that were significantly enriched within the avian missing gene data. The significance of each biological/disease or canonical pathway was further tested by Fisher’s Exact Test (α = 0.05).

To further confirm that disease states and pathways discovered by IPA were specifically associated with the missing gene set, and not present in any randomly selected set of genes we conducted additional IPA analyses on two sets of independently derived control gene sets consisting of 274 genes. To construct these sets, we first retrieved the entire set of protein coding genes from Ensembl (e71) that corresponded to the complete collections of 1-to-1 orthologs in human and lizard, and sorted them according to human chromosomal gene order. Custom scripts were written in R [57]; ‘Missing_gene_analysis.R’ is available at [58]) to generate control sets that contained blocks of genes (syntenic orthologs) with the same relative size (in Mb), number of genes, and chromosomal distribution as the avian missing blocks presented in Additional file 1: Table S1A). For each block, consisting of N genes (in blocks or as a singleton) on a specific chromosome, we randomly selected a ‘seed’ gene from that chromosome using the pseudo random number generator function supplied with statistical package R (RNG.kind = ‘Mersenne-Twister’; [59]). We then confirmed that all N genes were indeed on the correct chromosome, and were in the same syntenic gene order in both humans and lizard.

In a separate analysis, we also retrieved the sets of phenotypes associated with spontaneous, induced or genetically-engineered mutations in genes on our missing gene list, based on the Mouse Genome Informatics (MGI; [22]) database. We then classified the retrieved entries according to the affected tissues or organ systems (Additional file 1: Table S10A). We note that we only retrieved phenotypes where the deletion of a single gene is sufficient for the phenotype to be observed. We also used MGI to identify a subset of missing genes that are associated with a lethal phenotype (including partial and complete embryonic or perinatal lethal, or premature death) in rodents. We next examined the retrieved entries individually to identify cases where knockout of the gene of interest is sufficient for the lethal phenotype (Additional file 1: Table S11A) vs. cases where a combined knockout of one or additional genes is required for lethality (Additional file 1: Table S11B). We also used the MGI database, consultations to the OMIM [23], and keyword searches of Entrez Gene summaries (with terms such as syndrome, disease, mutation, deletion, or loss) to identify gene sets that are associated with genetic disorders in humans. Among these, we manually verified individual OMIM entries searching for evidence of diseases caused by loss of gene or gene function (Additional file 1: Table S12A in; typically autosomal recessive disorders, but also including cases of X-linked disorders or autosomal dominant haploinsufficiency) in contrast to disorders caused by gain of function mutations (Additional file 1: Table S12B).

To determine whether the associations with severe and/or lethal phenotype in mice were unique to missing genes, or more generally associated with any comparably sized gene sets, we also performed a complete MGI phenotype classification on 1,000 independent permutations of 274 genes. Using the control set algorithm described above for the IPA, we constructed 1,000 control gene lists and then for each list, retrieved sets of MGI phenotypes that were associated with each gene. Note that the phenotype, ‘No abnormal phenotype detected’ was not included in the analysis. A two-sided permutation test (α = 0.05) was used to test for differences between the number of phenotypes associated with the missing gene set versus the distribution of the number of phenotypes associated with 1,000 permuted control sets (Additional file 4: Figure S3). We also compared the number of genes associated with each phenotype in the missing gene set versus the distribution of the number of genes associated with the same phenotype in the permutation gene sets using a two-sided permutation test with Benjamin-Hochberg false discovery rate (FDR) multiple comparison correction (Additional file 1: Table S10B).

To determine whether the associations of missing genes with OMIM disease terms was greater (or less) than what would expected by chance, we analyzed the distribution of OMIM disease terms associated with the same 1,000 control gene sets described above for the MGI mouse phenotype analysis. Two-tailed permutation testing (α = 0.05) was used to test for statistical differences between the number of genes associated with an OMIM disease term in the missing gene set vs. the distribution of the numbers of disease terms associated with 1,000 control sets (Additional file 4: Figure S3).

To compare the impact of the same set of avian missing genes against the genetic backgrounds of chicken, humans, and lizard we conducted a comparative functional enrichment analysis. For each species (chicken, humans, and lizard), we first retrieved nucleotide sequences corresponding to the full set of Ensembl (e71) predicted transcripts, selecting the largest open reading frame for each gene. We then BLAST-aligned (BLAST) each sequence against NCBI’s non-redundant protein database (BLAST Expected value = 1.0E-3; matrix = BLOSUM62). We then used Blast2GO ([24]) to extract Gene Ontology (GO) terms associated with each NCBI hit (E-Value Hit Filter = 1.0E-6; Annotation cutoff = 55; GO-Weight = 5; HSP-Hit Overlap = 0) in order to identify the top 20 most similar protein coding sequences. Based on these alignments, we then used to Blast2GO to assign a set of evaluated GO annotations to each query sequence. Each nucleotide sequence was also subjected to protein domain motif scanning (Interpro scan) in Blast2GO, and the resulting additional GO annotations were merged with the Blast2GO annotations. We observed that the average number of annotations obtained per genome was in the 80,000 to 130,000 range, and was comparable across species. Finally, for each species, we performed a pairwise comparison of GO terms associated with the missing gene set vs. those associated with the remaining protein coding genes, and using a two-tailed Fisher’s exact test (α = 0.05) identified GO terms enriched in the missing gene set (Additional file 1: Table S14). We note that for the chicken comparison we used the missing gene set created for the lizard comparison.

To identify GO term enrichments that were unique or shared within the pairwise comparisons performed in chicken, lizard, and humans we used a Venn diagram (Venny; [60]). We specifically identified significantly enriched terms that were: A, enriched in the non-avian species (lizard/human), but not birds (Figure 7, Group A, yellow panels); B1, enriched only in chicken (Group B1, dark blue); B2, enriched in birds and humans and/or lizard (green); or C, enriched in chicken and lizard (gray). For each of these groups we then retrieved the corresponding sets of genes that were associated with each Groups (A to C) statistically enriched set of GO terms. In some instances, we found that a gene associated with one set of GO enrichments in a group (for example, Group A) was associated with a different set of GO enrichments in a different group (for example, Group B1), creating a potential conflict. To resolve these conflicts we retrieved from each group the corresponding sets of GO terms associated with the gene of interest, and evaluated whether the GO terms were descriptively similar (for example, protein kinase activity vs. kinase activity), or referred to very different functions and/or processes. For cases where the GO terms described a similar function, we used a conservative interpretation, and placed the gene in the category with the most inclusive species membership. In contrast, if the GO terms referred to very different functions, indicating the possibility that protein coding domains within the same protein might be differentially compensated across lineages, we included the gene in each group. The results of this analysis and classification are present in Additional file 1: Table S15.

We note that recent papers have pointed to some limitations when performing comparative analyses of functional GO annotations and enrichments, particularly when in the context of identifying orthologous vs. paralogous genes across lineages (for example, [61,62]). However, despite these limitations, functional GO enrichment analysis remains arguably the best approach currently available for comparative analysis with species other than mouse or humans. Unlike other functional enrichment analyses that rely heavily on existing gene curation (for example, DAVID, IPA), Blast2GO treats each gene as if it was a ‘novel gene’, and uses BLAST to annotate each novel gene sequence based on the presence of known protein motifs. The motif annotations are then represented by a universal set of Gene Ontology Terms. Although there may be some limitations to this approach due in large part to the limited availability of non-mammalian databases of annotated protein sequences, it still provides the best available tool for attempting to functionally annotate genes in non-rodent and non-human species. Moreover, because this method compares actual vs. theoretical losses within each organism’s background genome, we were able to further minimize biases due to differences in the overall genomic background of the species being compared.

## Additional files

Additional file 1: Table S1. Missing genes in syntenic blocks, or in close proximity to syntenic blocks, ordered according to chromosomal location in lizard. **Table S2.** Curation of misannotated Ensembl, Entrez Gene, and RefSeq genes in birds. **Table S3.** Evidence for closely related avian paralogs of missing genes in birds. **Table S4.** Evidence supporting the presence of genes in avian Entrez gene, RefSeq, and cloned mRNA databases, as well as lizard/human mRNA and protein BLAT/BLAST searches of avian genomes, trace archives, and EST/mRNA databases. **Table S5.** Annotation of false positive lizard Ensemble model BLAT alignments to the chicken genome. **Table S6.** Genes not found in chicken, but possibly present in other birds based on RefSeq annotations and on tBLASTn Searches of 60 Avian WGS contigs. **Table S7.** Genes previously reported as missing in birds. Reference citations are presented in Additional file 5. **Table S8.** Assessment of the presence of avian missing genes in crocodilian genomes. **Table S9.** Detailed list of functions associated with functional enrichment categories presented in Table 2A. **Table S10.** Major organs and systems affected by the loss of the missing avian genes in mice. **Table S11.** Genes whose deletion is associated with traits/phenotypes affecting tissues or organs that are absent in birds. **Table S12.** Genes that result in lethality alone in knockout mice, or when combined with other genes. **Table S13.** Genes associated with human disease and/or syndromes. **Table S14.** Gene Ontology terms that are enriched (BLAST2GO; *P* <0.05) in the chicken, human, and/or lizard lineages. **Table S15.** Possible functional consequences (and compensations) for genes associated with lethal and disease phenotypes in mammals. Reference citations are present in Additional file 5. **Table S16.** Human tissue specific expression of avian missing genes. **Table S17.** Orthogroup analysis of the avian missing genes presented in the Blast2Go analysis presented in Table S15. **Table S18.** Avian genes on unplaced chromosomes representing partial sequences with no synteny verification. Additional file 2: Figure S1. Avian missing syntenic blocks and chromosomal rearrangements. The avian missing syntenic blocks are closely associated with **(A)** inter- and **(B, C)** intra-chromosomal rearrangements that are revealed by local chromosomal alignments of 1-to-1 orthologous genes in chicken and humans. Orthologs are aligned according to human chromosome location. Syntenically ordered genes that are missing in birds (that is, chicken) are shaded in orange or gray (as Additional file 1: Table S1); flanking genes that are present in chicken, humans, and lizard (not shown) are shown in white. The position of each gene locus is indicated by chromosome number (for example, chr2, 19) and the start and end base for each corresponding Ensembl gene model. The location of several orthologous blocks that were removed for clarity is indicated by the dotted lines beneath the gene start/end columns. The solid line in C separates two adjacent syntenic blocks that are found on different chromosomal segments in lizard, and thus do not constitute a single block. In (A), the locations of the syntenic blocks in chicken that immediately flank the missing gene block are on different chromosomes (that is, chr4 and chrZ). In (B) and (C), the flanking blocks are on the same chromosomes, but are out of order (B), or several megabases apart (C), in comparison to their location in humans. Additional file 3: Figure S2. Analysis of gene size and protein sequence divergence for the avian missing gene set. Description of data: **(A)** Frequency distributions of predicted protein sizes for lizard orthologs of the avian missing genes and the entire set of lizard genes that is present in birds. The overall distributions of predicted protein size are similar. The relative percentage of short genes (that is, <500 bp) is also comparable across the two gene sets (9% vs. 10%). **(B)** The sizes of each missing gene are plotted against the percent amino acid identity (% AA Identity) of orthologous predicted proteins in humans vs. lizard. Additional file 4: Figure S3. MGI mouse phenotype and OMIM disease term analysis. Description of data: Plots showing distributions of the numbers of genes associated with MGI mouse phenotypes **(A)**, or OMIM disease terms **(B)** for 1,000 independently derived control gene sets. The average number of phenotypes (A) or disease terms (B) associated with the control gene sets is indicated by the blue lines; the number of phenotypes (A) and disease terms (B) associated with the missing gene set is indicated by the red lines. Two-tailed permutation tests reveal that the number of genes associated with both mouse phenotypes and OMIM disease terms is significantly less than that associated with the control gene sets (*P* = 0.001 and *P* = 0.02, respectively). (PDF 582 kb) Additional file 5: Figure S4. Distribution of mouse phenotypes in avian missing and control gene sets. Description of data: Plot showing the relative distribution of avian missing genes (black bars) vs. 1,000 permuted control genes (white bars; error bars denote standard deviation) for the set of mouse phenotypes (n = 32) that was associated with at least four genes (see Methods for details). Corresponding mouse phenotypes and phenotype descriptions for each phenotype number can be found in Additional file 1: Table S10B (listed in column A). Additional file 6:
**Supplemental literature cited.** References cited in Additional file 1: Tables S7 and S15.
