# Supplementary material for: Conserved syntenic clusters of protein coding genes are missing in birds
Source: Genome Biol. 2014 Dec 18;15(12):565. doi: 10.1186/s13059-014-0565-1 (PMC4290089; doi:10.1186/s13059-014-0565-1)
Supplement: Additional file 2: Figure S1. — Avian missing syntenic blocks and chromosomal rearrangements. The avian missing syntenic blocks are closely associated with (A) inter- and (B, C) intra-chromosomal rearrangements that are revealed by local chromosomal alignments of 1-to-1 orthologous genes in chicken and humans. Orthologs are aligned according to human chromosome location. Syntenically ordered genes that are missing in birds (that is, chicken) are shaded in orange or gray (as Additional file 1: Table S1); flanking genes that are present in chicken, humans, and lizard (not shown) are shown in white. The position of each gene locus is indicated by chromosome number (for example, chr2, 19) and the start and end base for each corresponding Ensembl gene model. The location of several orthologous blocks that were removed for clarity is indicated by the dotted lines beneath the gene start/end columns. The solid line in C separates two adjacent syntenic blocks that are found on different chromosomal segments in lizard, and thus do not constitute a single block. In (A), the locations of the syntenic blocks in chicken that immediately flank the missing gene block are on different chromosomes (that is, chr4 and chrZ). In (B) and (C), the flanking blocks are on the same chromosomes, but are out of order (B), or several megabases apart (C), in comparison to their location in humans. [file 13059_2014_565_MOESM2_ESM.pdf]

A. Interchromosomal Rearrangement

Gene Name	Chicken Ensembl Gene ID	Chicken Chr	Chicken Gene Start (bp)	Chicken Gene End (bp)	Human Ensembl Gene ID	Human Chr	Human Gene Start (bp)	Human Gene End (bp)
TRABD2A	ENSGALG00000015337	Z	51,788,745	52,080,838	ENSG00000186854	2	85,048,774	85,134,132
MAT2A	ENSGALG00000015366	Z	53,262,872	53,287,724	ENSG00000168906	2	85,766,288	85,772,403
RNF181					ENSG00000168894	2	85,822,848	85,824,704
TMEM150A					ENSG00000168890	2	85,825,671	85,829,821
ATOH8	ENSGALG00000020110	4	88,543,366	88,543,635	ENSG00000168874	2	85,978,467	86,015,189
IMMT	ENSGALG00000015783	4	88,714,416	88,727,264	ENSG00000132305	2	86,371,055	86,422,893
REEP1	ENSGALG00000015786	4	88,752,157	88,768,328	ENSG00000068615	2	86,441,116	86,565,206

B. Intrachromosomal Rearrangement

Gene Name	Chicken Ensembl Gene ID	Chicken Chr	Chicken Gene Start (bp)	Chicken Gene End (bp)	Human Ensembl Gene ID	Human Chr	Human Gene Start (bp)	Human Gene End (bp)
RNPS1	ENSGALG00000009288	14	14,582,361	14,588,230	ENSG00000205937	16	2,303,117	2,318,413
NTN3	ENSGALG00000009208	14	14,766,050	14,782,217	ENSG00000162068	16	2,521,500	2,524,146
PDPK1	ENSGALG00000006418	14	7,390,284	7,420,767	ENSG00000140992	16	2,587,965	2,653,189
KCTD5	ENSGALG00000006423	14	7,420,918	7,445,316	ENSG00000167977	16	2,732,476	2,759,031
KREMEN2					ENSG00000131650	16	3,013,945	3,018,381
PKMYT1					ENSG00000127564	16	3,018,025	3,030,540
PAQR4					ENSG00000162073	16	3,019,246	3,023,490
CCDC64B					ENSG00000162069	16	3077683	3,086,927
MMP25					ENSG00000008516	16	3096682	3,110,727
TRAP1	ENSGALG00000007686	14	13,176,992	13,195,178	ENSG00000126602	16	3,701,640	3,767,598
CREBBP	ENSGALG00000007762	14	13,198,040	13,266,202	ENSG00000005339	16	3,775,055	3,930,727
MGRN1	ENSGALG00000007616	14	13,016,046	13,094,671	ENSG00000102858	16	4,666,494	4,740,975
ANKS3	ENSGALG00000000643	14	13,552,792	13,573,785	ENSG00000168096	16	4,746,513	4,784,379

C. Intrachromosomal Rearrangement

Gene Name	Chicken Ensembl Gene ID	Chicken Chr	Chicken Gene Start (bp)	Chicken Gene End (bp)	Human Ensembl Gene ID	Human Chr	Human Gene Start (bp)	Human Gene End (bp)
VMA21	ENSGALG00000009065	4	17,648,229	17,653,101	ENSG00000160131	X	150,564,987	150,577,836
PRRG3	ENSGALG00000007290	4	11,020,781	11,022,731	ENSG00000130032	X	150,863,596	150,874,396
GABRA3	ENSGALG00000007269	4	10,844,309	10,892,925	ENSG00000011677	X	151,334,706	151,620,337
ZNF185	ENSGALG00000007488	4	11,312,405	11,338,388	ENSG00000147394	X	152,082,986	152,142,025
HAUS7					ENSG00000213397	X	152,713,124	152,760,978
BGN					ENSG00000182492	X	152,760,397	152,775,012
ATP2B3					ENSG00000067842	X	152,783,134	152,848,397
DUSP9					ENSG00000130829	X	152,907,946	152,916,781
PNCK					ENSG00000130822	X	152,935,185	152,954,465
SLC6A8					ENSG00000130821	X	152,953,554	152,962,048
ABCD1					ENSG00000101986	X	152,991,272	152,995,178
PLXNB3					ENSG00000198753	X	153,029,651	153,044,801
TMEM187					ENSG00000177854	X	153,237,778	153,248,646
IRAK1					ENSG00000184216	X	153,275,951	153,285,431
ATP6AP1					ENSG00000071553	X	153,656,978	153,664,862
GDI1					ENSG00000203879	X	153,665,266	153,671,814
PLXNA3					ENSG00000130827	X	153,686,621	153,701,989
LAGE3					ENSG00000196976	X	153,705,241	153,707,596
UBL4A					ENSG00000102178	X	153,712,056	153,714,932
SLC10A3					ENSG00000126903	X	153,715,645	153,719,016
DKC1	ENSGALG00000005054	4	2,098,487	2,106,571	ENSG00000130826	X	153,991,031	154,005,964
MPP1	ENSGALG00000005071	4	2,107,568	2,122,122	ENSG00000130830	X	154,006,959	154,049,282
F8	ENSGALG00000005077	4	2,126,164	2,145,986	ENSG00000185010	X	154,064,063	154,255,215
FUNDC2	ENSGALG00000005381	4	2,151,835	2,155,834	ENSG00000165775	X	154,254,255	154,288,578
