# Supplementary figures and images for: Conserved syntenic clusters of protein coding genes are missing in birds
Source: Genome Biol. 2014 Dec 18;15(12):565. doi: 10.1186/s13059-014-0565-1 (PMC4290089; doi:10.1186/s13059-014-0565-1)

**A**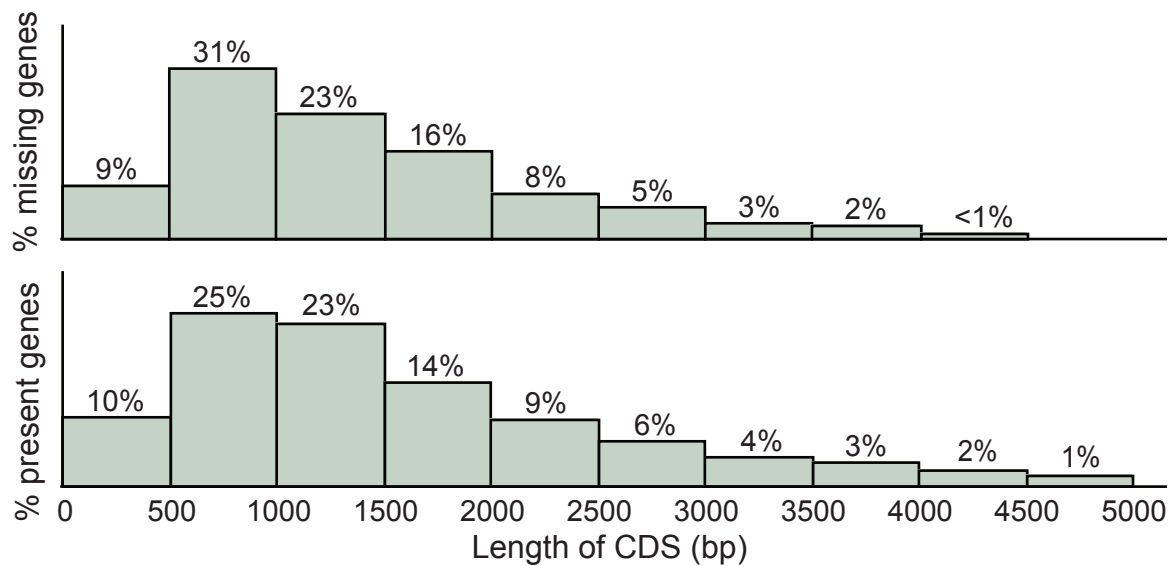**B**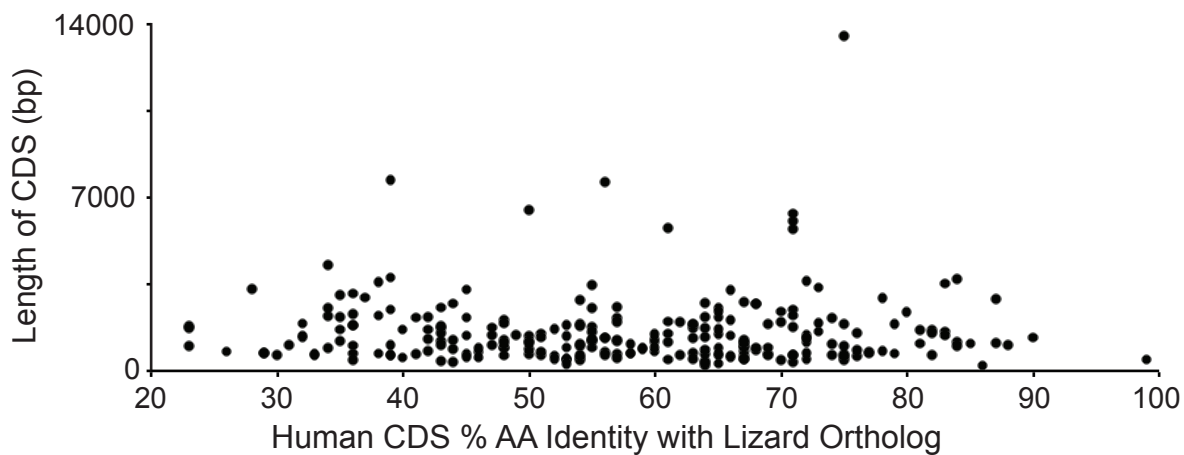

Supplement: Additional file 3: Figure S2. — Analysis of gene size and protein sequence divergence for the avian missing gene set. Description of data: (A) Frequency distributions of predicted protein sizes for lizard orthologs of the avian missing genes and the entire set of lizard genes that is present in birds. The overall distributions of predicted protein size are similar. The relative percentage of short genes (that is, <500 bp) is also comparable across the two gene sets (9% vs. 10%). (B) The sizes of each missing gene are plotted against the percent amino acid identity (% AA Identity) of orthologous predicted proteins in humans vs. lizard. [file 13059_2014_565_MOESM3_ESM.pdf]

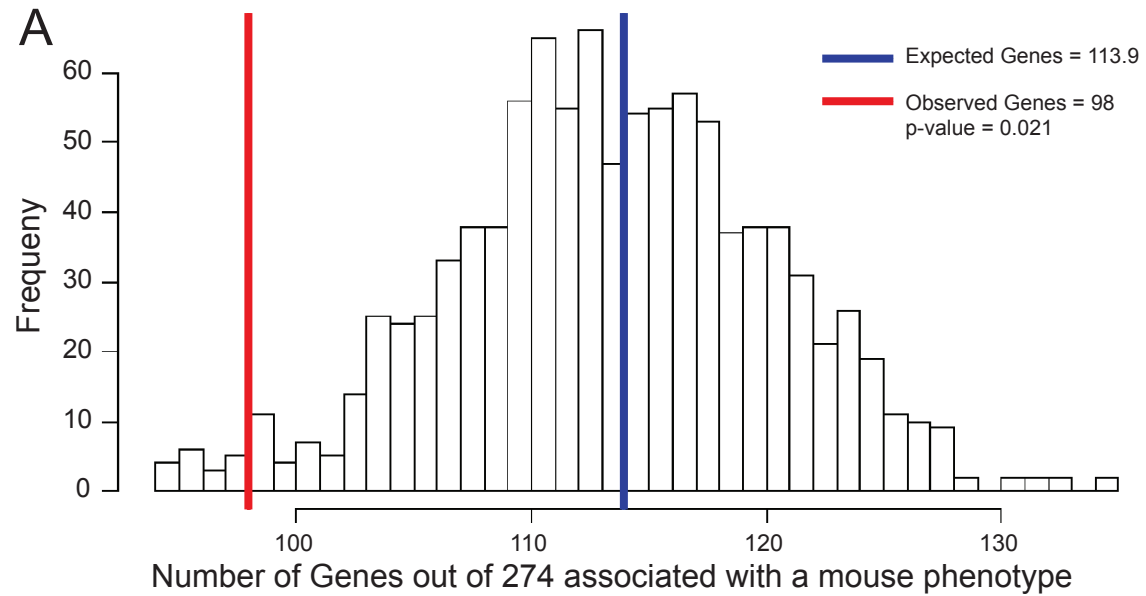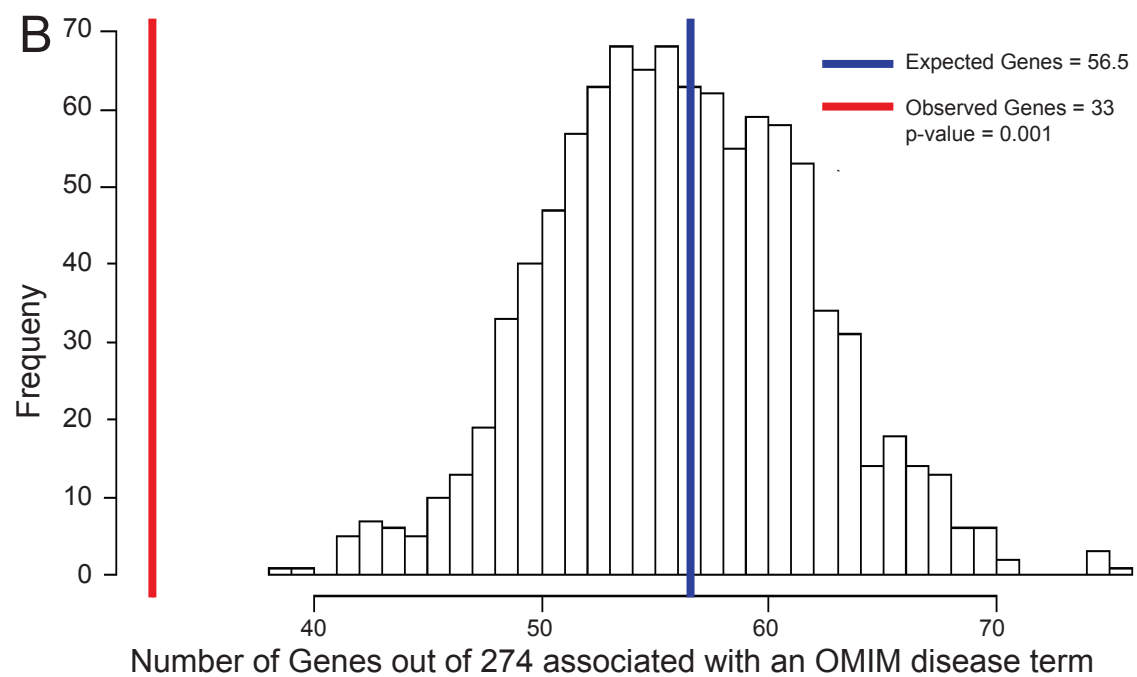

Supplement: Additional file 4: Figure S3. — MGI mouse phenotype and OMIM disease term analysis. Description of data: Plots showing distributions of the numbers of genes associated with MGI mouse phenotypes (A), or OMIM disease terms (B) for 1,000 independently derived control gene sets. The average number of phenotypes (A) or disease terms (B) associated with the control gene sets is indicated by the blue lines; the number of phenotypes (A) and disease terms (B) associated with the missing gene set is indicated by the red lines. Two-tailed permutation tests reveal that the number of genes associated with both mouse phenotypes and OMIM disease terms is significantly less than that associated with the control gene sets (P = 0.001 and P = 0.02, respectively). (PDF 582 kb) [file 13059_2014_565_MOESM4_ESM.pdf]

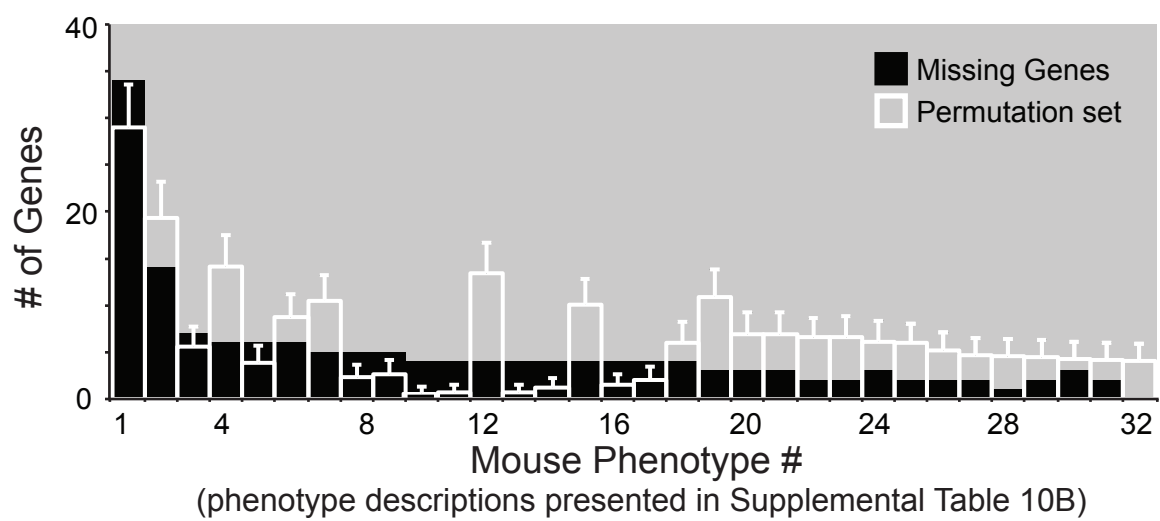

Supplement: Additional file 5: Figure S4. — Distribution of mouse phenotypes in avian missing and control gene sets. Description of data: Plot showing the relative distribution of avian missing genes (black bars) vs. 1,000 permuted control genes (white bars; error bars denote standard deviation) for the set of mouse phenotypes (n = 32) that was associated with at least four genes (see Methods for details). Corresponding mouse phenotypes and phenotype descriptions for each phenotype number can be found in Additional file 1: Table S10B (listed in column A). [file 13059_2014_565_MOESM5_ESM.pdf]
