# Supplementary material for: Conserved syntenic clusters of protein coding genes are missing in birds
Source: Genome Biol. 2014 Dec 18;15(12):565. doi: 10.1186/s13059-014-0565-1 (PMC4290089; doi:10.1186/s13059-014-0565-1)
Supplement: Additional file 6: — Supplemental literature cited. References cited in Additional file 1: Tables S7 and S15. [file 13059_2014_565_MOESM6_ESM.docx]

**Supplemental literature cited***

Ahn D, You KH, Kim CH (2012) Evolution of the tbx6/16 subfamily genes in vertebrates: insights from zebrafish. Mol Biol Evol 29: 3959-3983.

Alon LT, Pietrokovski S, Barkan S, Avrahami L, Kaidanovich-Beilin O, et al. (2011) Selective loss of glycogen synthase kinase-3alpha in birds reveals distinct roles for GSK-3 isozymes in tau phosphorylation. FEBS Lett 585: 1158-1162.

Blaschke UK, Hedbom E, Bruckner P (1996) Distinct isoforms of chicken decorin contain either one or two dermatan sulfate chains. J Biol Chem 271: 30347-30353.

Braun EJ, Sweazea KL (2008) Glucose regulation in birds. Comp Biochem Physiol B Biochem Mol Biol 151: 1-9.

Carre W, Wang X, Porter TE, Nys Y, Tang J, et al. (2006) Chicken genomics resource: sequencing and annotation of 35,407 ESTs from single and multiple tissue cDNA libraries and CAP3 assembly of a chicken gene index. Physiol Genomics 25: 514-524.

Christie GR, Williams DJ, Macisaac F, Dickinson RJ, Rosewell I, et al. (2005) The dual-specificity protein phosphatase DUSP9/MKP-4 is essential for placental function but is not required for normal embryonic development. Mol Cell Biol 25: 8323-8333.

Dolbeer R, Link M, Woronecki P (1988) Naphthalene shows no repellency for starlings. Wildl Soc Bull 16: 62-64.

Gutierrez-Caballero C, Herran Y, Sanchez-Martin M, Suja JA, Barbero JL, et al. (2011) Identification and molecular characterization of the mammalian alpha-kleisin RAD21L. Cell Cycle 10: 1477-1487.

Hätinen T, Holm L, Airaksinen MS (2007) Loss of neurturin in frog--comparative genomics study of GDNF family ligand-receptor pairs. Mol Cell Neurosci 34: 155-167.

Hoch RV, Rubenstein JL, Pleasure S (2009) Genes and signaling events that establish regional patterning of the mammalian forebrain. Semin Cell Dev Biol 20: 378-386.

Hultqvist G, Ocampo Daza D, Larhammar D, Kilimann MW (2012) Evolution of the vertebrate paralemmin gene family: ancient origin of gene duplicates suggests distinct functions. PLoS One 7: e41850.

Hutson S (2001) Structure and function of branched chain aminotransferases. Prog Nucleic Acid Res Mol Biol 70: 175-206.

Ka S, Markljung E, Ring H, Albert FW, Harun-Or-Rashid M, et al. (2013) Expression of carnitine palmitoyl-CoA transferase-1B is influenced by a cis-acting eQTL in two chicken lines selected for high and low body weight. Physiol Genomics 45: 367-376.

Lagerstrom MC, Hellstrom AR, Gloriam DE, Larsson TP, Schioth HB, et al. (2006) The G protein-coupled receptor subset of the chicken genome. PLoS Comput Biol 2: e54. 23.

Lardelli M (2003) The evolutionary relationships of zebrafish genes tbx6, tbx16/spadetail and mga. Dev Genes Evol 213: 519-522.

Lemoine M, Dupont J, Guillory V, Tesseraud S, Blesbois E (2009) Potential involvement of several signaling pathways in initiation of the chicken acrosome reaction. Biol Reprod 81: 657-665.

Littman MP, Wiley CA, Raducha MG, Henthorn PS (2013) Glomerulopathy and mutations in NPHS1 and KIRREL2 in soft-coated Wheaten Terrier dogs. Mamm Genome 24: 119-126.

Makarchikov AF, Lakaye B, Gulyai IE, Czerniecki J, Coumans B, et al. (2003) Thiamine triphosphate and thiamine triphosphatase activities: from bacteria to mammals. Cell Mol Life Sci 60: 1477-1488.

Minchin RF, Boyd MR (1983) Localization of metabolic activation and deactivation systems in the lung: significance to the pulmonary toxicity of xenobiotics. Annu Rev Pharmacol Toxicol 23: 217-238.

Miner JH (2012) Life without nephrin: it's for the birds. J Am Soc Nephrol 23: 369-371.

Shang P, Hoogerbrugge J, Baarends WM, Grootegoed JA (2013) Evolution of testis-specific kinases TSSK1B and TSSK2 in primates. Andrology 1: 160-168.

Simsa S, Genina O, Ornan EM (2007) Matrix metalloproteinase expression and localization in turkey (Meleagris gallopavo) during the endochondral ossification process. J Anim Sci 85: 1393-1401.

Skiba-Cassy S, Collin A, Chartrin P, Medale F, Simon J, et al. (2007) Chicken liver and muscle carnitine palmitoyltransferase 1: nutritional regulation of messengers. Comp Biochem Physiol B Biochem Mol Biol 147: 278-287.

Spicer AP, McDonald JA (1998) Characterization and molecular evolution of a vertebrate hyaluronan synthase gene family. J Biol Chem 273: 1923-1932.

Völker LA, Petry M, Abdelsabour-Khalaf M, Schweizer H, Yusuf F, et al. (2012) Comparative analysis of Neph gene expression in mouse and chicken development. Histochem Cell Biol 137: 355-366.

Whittle BJ, Hamid S, Lidbury P, Rosam AC (1985) Specificity between the anti-aggregatory actions of prostacyclin, prostaglandin E1 and D2 on platelets. Adv Exp Med Biol 192: 109-125.

Wu G, Chung-Bok MI, Vincent N, Kowalski TJ, Choi YH, et al. (1998) Distribution of phosphate-activated glutaminase isozymes in the chicken: absence from liver but presence of high activity in pectoralis muscle. Comp Biochem Physiol B Biochem Mol Biol 120: 285-290.

Xavier CP, Eichinger L, Fernandez MP, Morgan RO, Clemen CS (2008) Evolutionary and functional diversity of coronin proteins. Subcell Biochem 48: 98-109.

Yamamoto M, Meno C, Sakai Y, Shiratori H, Mochida K, et al. (2001) The transcription factor FoxH1 (FAST) mediates Nodal signaling during anterior-posterior patterning and node formation in the mouse. Genes Dev 15: 1242-1256.

* References cited in Tables S7 and S15 in Additional file 1.
